# Ferrocene-Based Drugs, Delivery Nanomaterials and Fenton Mechanism: State of the Art, Recent Developments and Prospects †

**DOI:** 10.3390/pharmaceutics15082044

**Published:** 2023-07-29

**Authors:** Catia Ornelas, Didier Astruc

**Affiliations:** 1 ChemistryX, R&D Department, R&D and Consulting Company, 9000-160 Funchal, Portugal; 2 University of Bordeaux, ISM, UMR CNRS, No. 5255, 351 Cours de la Libération, CEDEX, 33405 Talence, France

**Keywords:** ferrocene, metallodrug, anticancer drug, cancer, parasitic, bacterial, fungal and viral diseases, drug delivery, Fenton reaction, hydroxyl radical

## Abstract

Ferrocene has been the most used organometallic moiety introduced in organic and bioinorganic drugs to cure cancers and various other diseases. Following several pioneering studies, two real breakthroughs occurred in 1996 and 1997. In 1996, Jaouen et al. reported ferrocifens, ferrocene analogs of tamoxifen, the chemotherapeutic for hormone-dependent breast cancer. Several ferrocifens are now in preclinical evaluation. Independently, in 1997, ferroquine, an analog of the antimalarial drug chloroquine upon the introduction of a ferrocenyl substituent in the carbon chain, was reported by the Biot-Brocard group and found to be active against both chloroquine-sensitive and chloroquine-resistant strains of *Plasmodium falciparum*. Ferroquine, in combination with artefenomel, completed phase IIb clinical evaluation in 2019. More than 1000 studies have been published on ferrocenyl-containing pharmacophores against infectious diseases, including parasitic, bacterial, fungal, and viral infections, but the relationship between structure and biological activity has been scarcely demonstrated, unlike for ferrocifens and ferroquines. In a majority of ferrocene-containing drugs, however, the production of reactive oxygen species (ROS), in particular the OH. radical, produced by Fenton catalysis, plays a key role and is scrutinized in this mini-review, together with the supramolecular approach utilizing drug delivery nanosystems, such as micelles, metal–organic frameworks (MOFs), polymers, and dendrimers.

## 1. Introduction

The International Agency for Research on Cancer estimated that the number of cancer cases worldwide reached almost 20 million in 2020, with 10 million deaths [1]. Since conventional treatments, including surgery, radiotherapy, and chemotherapy, cannot cure all categories of cancer, a timely approach to treating solid tumors is the use of tumor-targeting drugs [2]. Likewise, there is an urgent need for environment purification and localized antibacterial treatment because bacterial infections represent the third cause of death worldwide (just after cardiovascular diseases), with an estimated 13.7 million of infectious origin in 2019 [3]. More generally, infectious diseases, in which microorganisms mature or propagate in or on hosts, can be divided into four categories: parasitic (with malaria as the biggest group), bacterial, fungal, and viral. The history of infectious diseases is covered with entire populations being periodically decimated, mostly with pathogenic bacteria or viruses as main infectious agents, as dramatically illustrated by the Black Death epidemic in the 14th century that decimated between a third and half of the European population and by the recent coronavirus COVID-19 that killed around 15 million people in two years, according to the World Health Organization (WHO). The invention of penicillin by A. Fleming in 1928, followed by a variety of antibiotics, subsequently brought considerable relief in the last century. Presently, their excessive use, in particular the prophylactic antibiotic therapy, brings about a dangerous situation because of the adaptability of bacteria, which quickly develop resistance, including sharing of resistant genes between bacteria. Without effective drugs for the prevention and treatment of infections, the human ability to combat common infectious diseases is threatened.

Nanomedicine should largely improve therapeutic strategies using sophisticated targeting of multifunctional drugs. Among a variety of tools, ferrocene chemistry benefits from both numerous possibilities of easy functionalization and stability of the Fe(II) and Fe(III) forms [4] with their easy interconversion in biological media at a mild redox potential [5]. Therefore, this great flexibility of design and physical properties of ferrocene-based drugs should be adaptable to the delicate criteria toward precise and efficient localized chemotherapy. In this mini-review, we will first briefly summarize seminal, early, and recent medical studies utilizing ferrocene-based pharmacophores. Then, we will focus attention on curing strategies utilizing the ferrocene redox system engaging radical mechanisms that involve Fenton chemistry in the production of reactive oxygen species (ROS), in particular OH. radicals. Such ROS provoke disruption of cellular processes via nonspecific attack of proteins, lipids, and DNA. In order to transport drugs, ferrocene-containing nanodrug cargos that can deliver systems onto sick cells, such as micelles, metal–organic frameworks (MOFs), and metallomacromolecules, including polymers and dendrimers, will be discussed. More than a thousand articles and numerous reviews have been published on the anticancer and other biomedical activities of ferrocene-based compounds [6,7,8,9,10,11,12,13,14,15,16,17,18,19,20,21,22,23,24,25,26,27,28,29,30,31,32,33].

## 2. Pioneering and Early Studies

**Figure 1:**
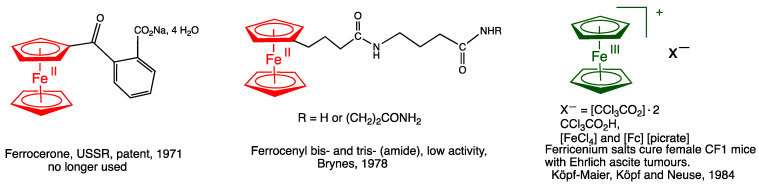
Early finding of the antitumoral properties of ferrocene derivatives and ferricenium salts.

Following a patent from a Soviet Union chemist on ferrocerone in 1971 (no longer in use), in 1976, Brynes et al. discovered the weak antitumor activity against lymphocytic leukemia P-388 of ferrocenyl derivatives containing a substituent that bears amide groups [34]. In 1984, some ferricenium salts were found by Köpf-Maier, Köpf, and Neuse to cure female mice with activity against Ehrlich ascites tumor (EAT) [35] (Figure 1).

It was only more than a decade later that the formation of DNA-damaging OH^.^ radicals from cytotoxic ferricenium and ferrocene derivatives was observed under physiological conditions [36,37]. Since then, the anticancer and antimicrobial activities of ferrocene-containing compounds using OH^.^ radicals generated by ferrocene-based drugs have been the subject of intense scrutiny.

**Scheme 1:**
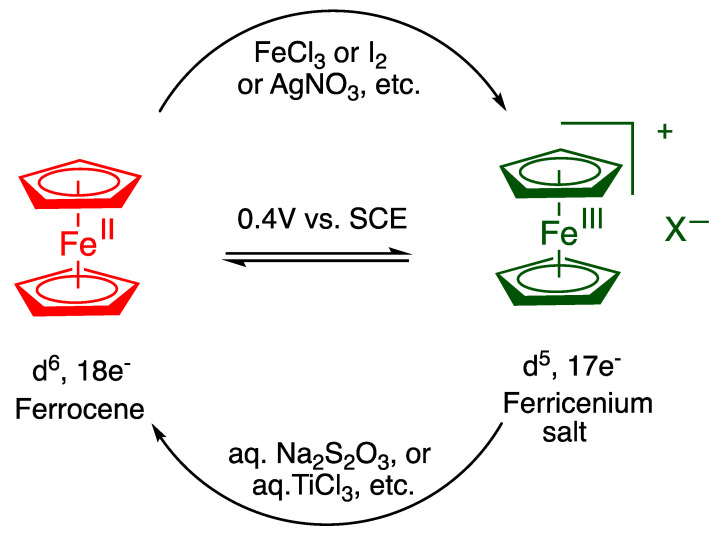
Ferrocene/ferricenium redox system: redox exchange using common redox reagents.

Two properties have appeared essential toward biological activity: (i) the drug delivery design and property of the medicinal cargo [38,39,40,41], and (ii) the chemical and electrochemical reversibility provided by the ferrocene/ferricenium redox system (Scheme 1), such as an optimal electron reservoir [42,43,44].

At an early stage in the field, Neuse, with Köpf-Maier and Köpf, observed activity against Ehrlich ascites tumor (EAT) of some simple water-soluble ferricenium salts (but not all) [44], and with Kanzawa, by in vitro human tumor clonogenic assay, he observed the metabolic oxidation of ferrocene derivatives to ferricenium equivalents in the cell [44]. These seminal works showed that even if the lipophilic sandwich ligands ensured the penetration through biological membranes, the water solubility was very important to the distribution of the drug into the organism. Interestingly, both Fe(III) and Fe(II) derivatives (although not ferrocene by itself) could exhibit antitumor properties.

A very wide variety of ferrocene compounds were reported during the last decade of the last century and the first decade of this century, showing antitumoral properties. These families of active ferrocene derivatives were reviewed in Ornelas’ 2011 perspective article [8]. Following Langer’s powerful concept of drug delivery [45,46], later emphasized in a review on cancer therapy in 2007 [47], Neuse applied this principle to polyaspartamide–ferrocene conjugates in 1998 [48]. A decade later, the first studies appeared with lipid nanocapsules [8,49] and cyclodextrin complexes (vide infra) [8,50].

On the side of antibacterial, antiparasitic, and antifungal ferrocene-based drugs, several studies appeared near the end of the last century. Epton and Marr et al. reported, in the 1970s, ferrocene analogs of penicillin and cephalosporins, in which the conventional phenyl or heteroaromatic group was replaced by a ferrocene moiety, with antibacterial activity sometimes comparable to that of benzyl-penicillin but lower than that of penicillin [51]. In 1993, Scutaru’s group reported activities toward Gram-positive bacteria of ferrocene-containing thioglycolic acid S, the antibacterial activities being similar to those of amoxicillin, cephalothin, and carbenicillin [52].

In 1997, the Biot-Brocard group reported the ferrocene-chloroquine analog, ferroquine, obtained by substituting the carbon chain of the antimalarial agent chloroquine with a ferrocenyl unit, together with its antimalarial activity on mice infected with *Plasmodium berghei N*. and *Plasmodium yoelii NS*. This new drug was 22 times more potent against schizontocides than chloroquine in vitro against a drug-resistant strain of *P. falciparum* [53]. In 2000, the same group also reported the antifungal activity of a ferrocene-fluconazole analog [54]. During that same year, antibacterial properties against *Escherichia coli, Pseudomonas aeruginosa, Staphylococcus aureus*, and *Klebsiella pneumonae* were reported by Chohan and Praveen for symmetric 1,1′-ferrocen-derived Schiff-base ligands and their Co(II), Cu(II), Ni(II), and Zn(II) chelates, the latter performing better than the ligands [55]. During the first decade of the century, a study by the Metzler-Nolte group retained attention on antibacterial activities of metallocene-peptide bioconjugates, in particular [Fc-C(O)WRWRW-NH_2_] (Fc = ferrocenyl) that was effective against Gram-positive *Staphylococcus aureus* [25,29,32,34,35,56]. The interest in this study is that numerous plants, insects, and animals possess a wide variety of antibacterial peptides with very different sizes in their immune system. Such research on antibacterial metallodrugs is important because the development of antibiotic resistance to synthetic antibiotics is current, and bacteria are developing resistance very fast, including sharing of resistant genes between bacteria. Numerous ferrocene-appended pharmacophores were reported during the second decade of this century and were summarized in reviews by Patra and Gasser and by Kumar’s group in 2016 and 2018, respectively [10,39,56].

## 3. Antitumoral Ferrocenyl Conjugates

**Figure 2:**
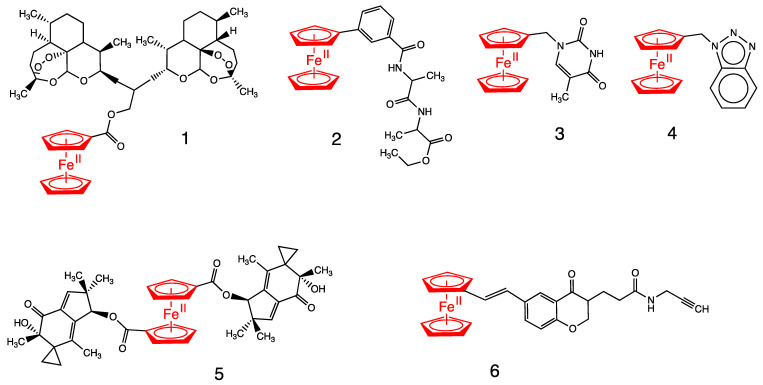
Chemical structures of typical examples of ferrocene-based pharmacophores with anticancer activity.

By far, the largest biomedical research activity utilizing the ferrocenyl moiety connected to bioactive molecules has concerned potential anticancer drugs. Artemisinins, peptides (lung cancer), nucleosides and nucleobases (against carcinoma 755), illudine (from mushrooms, extremely toxic), flavonoids, retinoid, curcumin (utilized in the Indian diet), and taxoid derivatives have been derived with ferrocene [12,30]. Ferrocene hybrid anticancer drug research included DNA-targeting compounds (DNA intercalator and inhibitors of topoisomerase II), breast cancer-targeting ferrocifen (prominent examples, analogs of the anti-breast cancer drug tamoxifen, largely developed by Jaouen, Vessières, and Top in Paris) [26,27], raloxifen complexes, prostate cancer-targeting androgens and anti-androgen ferrocenyl complexes, and ferrocenes attached to complexes of first raw (Fe, Co, Ni, Cu) or noble metals (Pt, Pd, Rh, Ir) [8]. Typical examples of ferrocene hybrid anticancer pharmacophores are compounds **1**–**6** shown in Figure 2.

The di-1,2,4-trioxane-ferrocene hybrid **1** is a very active artemisinin-ferrocene conjugate (IC_50_ = 0.01 μM) against the CCRF-CEM lymphoblastic leukemia cells [57,58]. The N-[meta-(ferrocenyl)benzoyl] dipeptide ethyl ester **2** shows very good antitumor action in vitro against H1299 human lung carcinoma cells with IC_50_ value of 26 μM [59]. Ferrocenyl bioconjugates of adenine, thymine, cytosine, and iodo-cytosine reported by Simenel, such as 1-N-ferrocenylmethyl thymine **3**, showed antitumoral effect against Ca755 cell line (murine adenocarcinoma) with 70% inhibition of tumor growth compared with the control [60,61]. Ferrocenylalkylbenzotriazole **4** showed up to 100% of tumor growth inhibition upon testing against solid tumors and achieved 45% regression. Bis(illudinyl M) 1,10–ferrocenedioate **5** incorporating illudin with ferrocene is not harmful to normal cells but more cell line-specific and highly cancer-selective compared with the parent illudin M [62]. Flavonoid compounds exhibited anticancer mechanisms, such as angiogenesis inhibition, inducing cell cycle. The ferrocene-flavonoid analog **6** (IC_50_: 23.0–35.0 μM) was sensitive to CCRF-CEM, MCF-7, and HepG2 cancer cell lines [63].

**Figure 3:**
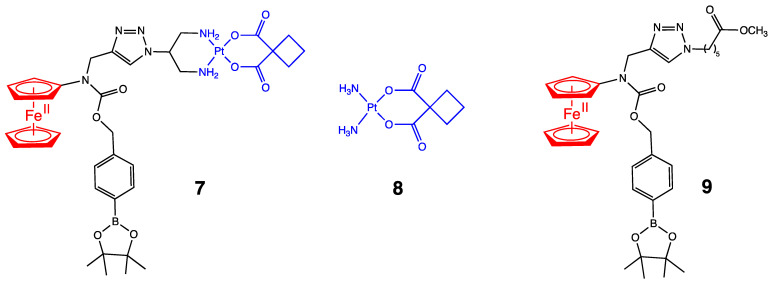
Chemical structures of triazole-containing ferrocenyl-carbopatin conjugate **7**, carboplatin **8**, and triazole-containing ferrocenyl-conjugate reference compound **9**.

The bioactivity of ferrocene-1,2,3-triazole conjugates, very recently scrutinized and reviewed by Koszytkowska-Stawińska and Buchowicz, revealed the interest in the 1,2,3-triazole link with ferrocene bioconjugates created by facile “click” chemistry from a synthetic point of view and also because the electronic delocalization of the ferrocenyltriazole provides redox potential modulation, thus increasing lipophilicity and π-π staking and hydrogen-bonding facilities that look favorable to bioactivity. These authors emphasized that several ferrocenyltriazole conjugates possessed anticancer properties of the same order of magnitude as those of ferrocifens [12]. Mokhir ‘s group found that conjugate **7** accumulated in the mitochondria of A2780 ovarian cancer cells more efficiently than the parent clinically approved drug carboplatin **8**. A synergistic effect between the “click”-linked carboplatin group of **7** and the other ferrocene-containing component was postulated since its activity was higher than that of an equimolar mixture of carboplatin **8** and reference compound **9** [64] (Figure 3).

Ferrocifens have been designed by Jaouen et al. [26] upon replacing, by a ferrocenyl group, a phenyl group of tamoxifen **10a** (Walpole, Richardson, 1962) and hydroxytamoxifen (TAM-OH, **10b**), which are selective estrogen receptor modulators (SERMs), decreasing the growth of breast cancer cells.

Tamoxifen is used for the treatment of both early and advanced estrogen receptor-positive (ER+) breast cancer [65] and presently is the most sold drug, even as a generic drug in some countries, against this cancer type, but its use suffers from contraindications and serious adverse side effects. Jaouen’s ferrocifen broad family of anticancer drugs derived from **11**, first reported in 1996 following synthesis via a tedious route [66], was shortly thereafter made readily accessible using the functional-group-tolerant McMurry coupling (Zn, TiCl_4_, THF) of two different ketones [67], propionyl ferrocene [26], FcC(O)Et, and a diaryl ketone, in particular di-(4-hydroxyphenyl) ketone, eventually followed by appropriate functionalization at the phenolic group [68]. Ferrocifens, such as **11** (R^1^ = R^2^ = OH) and Fc-Tam-OH **11** (R^1^ = OH, R^2^ = O(CH_2_)_3_N(CH_3_)_2_ are among the earliest organometallic selective estrogen receptor modulators (SERMs) known since they were described for the first time in 1996 [66]. Whereas tamoxifen shows antiestrogenic activity only on hormone-dependent MCF-7 breast cancer cells, ferrocifens, remarkably, are antiproliferative with dual effects on both hormone-dependent (MCF-7) and hormone-independent (MDA-MB-231) breast cancer cells (IC_50_ of Fc-OH-Tam: 0.8 and 0.5 mM, respectively). Relative binding affinity (RBA) on **11a** and **11b** and Fc-diOH showed at an early stage the possibilities of association with both *a* and *b* isoforms of the estrogen receptor. The antiproliferation effect of the ferrocifens shows that their cytotoxicity is much higher on cancer cells than on normal cells. The ferrocifens **11a** and **12a** were the most active against the NCI panel of 60 cell lines that represent nine cancer types (breast, leukemia, central nervous system, colon, kidney, lung, melanoma, ovary, and prostate) for which there are no satisfactory treatments [69]. The most active ferrocifens were **11a**, **11b** (the ferrocifen analog of TAM-OH), and the ansaferrocifen **12a** with, for instance, IC_50_ values on MDA-MB-231 cells (after 5 days of incubation) of 0.64, 0.5 and 0.089 mM, respectively [70]. Interestingly, the IC_50_ values obtained for these complexes were much lower than for their open-chain homologs, despite a less favorable conjugation of the ferrocene ring, due to the bent metallocene structure with the phenol-containing organic group [71]. Nevertheless, conjugation of the ferrocenyl moiety with the double bond plays an important role in optimizing cytotoxicity as, when this double bond is not conjugated to the ferrocenyl group, cytotoxicity becomes an order of magnitude lower. The ruthenocene and osmocene analogs of TAM-OH were also prepared and studied for their pharmacological properties, but they were less active than the corresponding ferrocifens, with their biological properties being essentially related to the amine chain, which emphasized the uniqueness of the ferrocifens [72].

**Figure 4:**
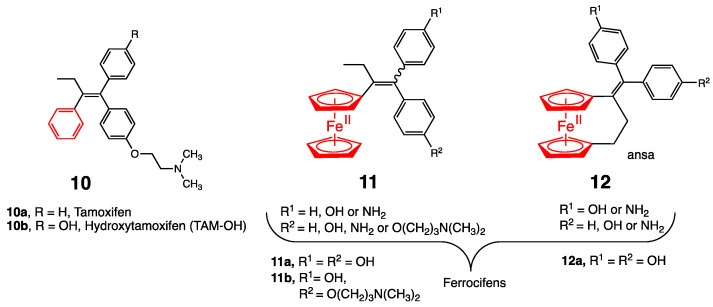
Chemical structures of tamoxifen and ferrocifen derivatives.

New ferrociphenols **13**–**15**, synthesized with a polar group on the alkyl chain, showed strong antiproliferative effects, especially against ovarian cancer cells that are resistant to cisplatin. Although, lengthening the ethyl chain with additional CH_2_ groups, which increases lipophilicity, resulted in decreased biological activity, introducing a hydroxy group on the propyl chain improved the activity of **13** against all the cell lines tested compared with **11a** [73,74]. With the larger aprotic imide ring group (succinimido in **14** or phthalimido in **15**), an even larger improvement in antitumoral activity on the three ovarian cancer cell lines, compared with **13**, was observed with IC_50_ values below 0.08 μM against A2780 cell line and cisplatin-resistant A2780-Cis cells, for **14** (Figure 4) [75].

The antiproliferative activity of several ferrocifen complexes, including **11a**, **11b**, and **13**, has been evaluated against 15 cell lines obtained from patients, against glioblastoma, the most aggressive type of brain cancer (glioma) (i.e., cancer cells divide very rapidly). These compounds showed a broad diversity of behavior. The study showed that **11a** activated the death receptor signaling pathway in sensitive patient-derived cell lines and acted via the modulation of the expression of FAS (a molecule involved in the regulation of cell death) [76].

**Figure 5:**
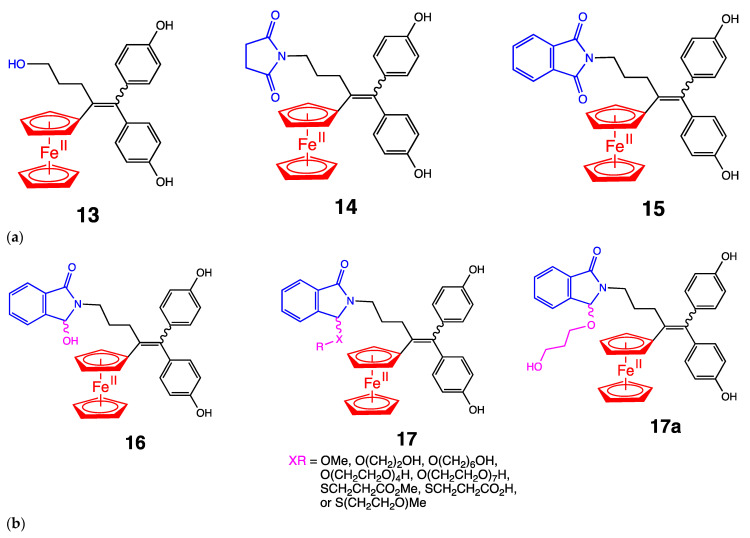
(**a**) Chemical structures of ferrociphenols **13**–**15**. (**b**) Chemical structure of derivatives of ferrociphenol **15** through reduction of one of the carbonyl groups of the imide groups to the corresponding α-hydroxylactam **16**, followed by conversion of the α-hydroxylactam **16** to highly electrophilic *N*-acyliminium ions with subsequent grafting of a wide variety of substituents.

Reduction of one of the carbonyl groups of the imide groups of **15** to the corresponding α-hydroxylactam **16** only slightly affected the antiproliferative activity. Conversion of α-hydroxylactam **16** to highly electrophilic *N*-acyliminium ions allowed grafting a wide variety of substituents to the polar imide motif of phthalimido ferrocidiphenol in **17**, which added polarity benefitting diverse cytotoxic activities on two breast cancer cell lines. The compound **17a**, i.e., **17** with XR = O(CH_2_)_3_OH, was the most active compound of the series and was selective against cancer cells (6- or 3-fold less toxic on hTERT-RPE1 with respect to MDA-MB-231 and MCF-7, respectively) (Figure 5) [76].

Most recent publications by Jaouen et al. report antiproliferative activity of ferrocifen derivatives against TNBC (triple-negative breast cancer) cells [77] and the inhibition of cathepsin B by ferrocenyl indenes [78].

Altogether, the design of ferrocifens has involved taking into account, in addition to the starting tamoxifen and tamoxifen-OH pharmacological properties, utilization of the extraordinary flexible ferrocene functionalization and introduction of additional key parameters, such as the refinement of reversible redox site adequate potential for OH. radical generation, the lipophilicity and bulk of the ferrocenyl group (the hydrophobic ferrocenyl group strongly binds the enzyme hydrophobic pockets, which causes inhibition), and solubilizing OH groups at various sites of the molecule, with the ability to provide important H-bonding capacities with the cell components. Over 25 years, Jaouen’s group has built up a group of more than 400 diverse ferrocifen derivatives optimizing major anticancer properties, some of which are in preclinical evaluation, and several offer other promising biological functions.

Many other ferrocene pharmacophores have been derived from the structures of natural products or drugs. Other drugs targeting breast cancer with SERMs are the ferrocenyl raloxifens, such as **19** (compare with raloxifen **18**), reported by Marques and showing cytotoxicity against ovarian, cervical, lung, colon, and breast cancer cell lines with IC_50_ values more than one order of magnitude lower than that of cisplatin, with apoptosis cell death mechanism and synergy in **19** between the raloxifen and ferrocenyl moieties [79].

**Figure 6:**
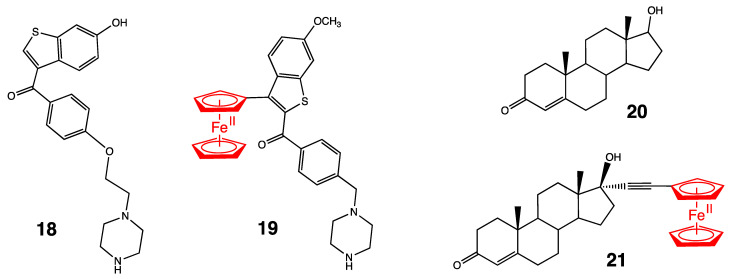
Chemical structures of raloxifen **18** and its ferrocenyl derivative **19**, and of dihydrotestosterone **20** and its ferrocenyl derivative **21**.

In the category of ferrocenyl androgens and antiandrogens targeting prostate cancer, the metabolite dihydrotestosterone **20**, of testosterone, promotes malignant prostate growth, but the ferrocenyl derivative **21** had low activity. Nevertheless, **21** presented strong antiproliferative activity on hormone-independent PC-3 prostate cancer cells with an IC_50_ value of 8.3 mM [80] (Figure 6).

Numerous steroid-ferrocene conjugates with variable anticancer properties have been synthesized and evaluated during the last 50 years, including both families of ferrocene-estrogen and ferrocene androgen conjugates, although the first systematic study of ferrocenyl and other organometallic steroidal androgens was reported by the Jaouen group in 2009 [80]. Other ferrocene estrogen [81] and androgen [82] conjugates were recently synthesized and evaluated by the Meléndez group, whereas the Sakač group recently reported new ferrocenyl estrogens [83] and reviewed the field [15].

Kumar’s group recently reviewed the advancements in anticancer hybrid drugs, including some ferrocene hybrids [14]. That two parts of a biomolecule exert two distinct functions in synergy toward anticancer properties was inherent to the concept of introduction of a ferrocene group into or branched to a drug in order to improve its antitumoral properties. The most recent examples are ferrocenyl pyrazoles [84] ferrocenyl chalcone amines [85] ferrocenyl chalcono (sugar) triazoles [86], and ferrocene coumarin hybrids [87], which all possess significant cytotoxic activities against various cell lines.

**Figure 7:**
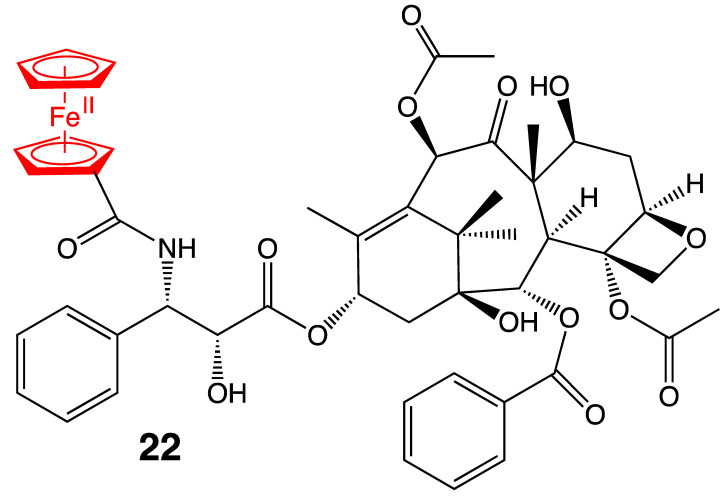
Chemical structure of the ferrocenyl derivative of Paclitaxel **22** that showed high anticancer activity.

In 2017, Rychlik’s group reported several ferrocenyl derivatives of paclitaxel, a diterpene antimitotic agent utilized against ovarian and breast carcinomas and Kaposi’s sarcoma. Antiproliferative activity was examined in the human tumor cell lines A549 (alveolar basal epithelial cell adenocarcinoma), COLO 205 (colorectal adenocarcinoma), HCT116 (colorectal adenocarcinoma), Hep G2 (hepatocellular carcinoma), MCF-7 (breast adenocarcinoma), and SW620 (colorectal adenocarcinoma), and efficiency was very dependent on the position of the ferrocenyl group in the molecule. The most promising compound was **22**, with IC_50_ = 0.005–0.0015 mM against SW620, A549, COLO 205, HCT116, Hep G2, and MCF-7 cells (Figure 7) [88].

Recently, several anticancer drugs were reported and evaluated, including aniline derivatives [32] and nucleoside analogs [89]. In the latter example, 1,3-disubstituted ferrocenes were much more cytotoxic than 1,1′-disubstituted isomers. The cytotoxic properties of quinolizidine alkaloids conjugated with ferrocene against cell lines HEK 293, Jurkat, A549, MCF-7, and SH-SY5Y were compared, and their activity against noncancerous HEK 293 cells was shown to be weak [90].

## 4. Antibacterial Ferrocenyl Conjugates

**Figure 8:**
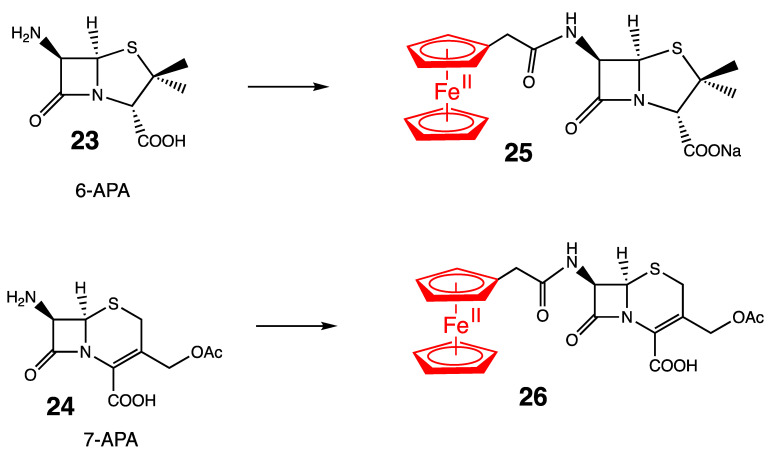
Chemical structures of the antibiotics 6-aminopenicillanic (6-APA) **23**, and 7-aminocephalosporanic acids **24**, and their ferrocenyl derivatives, the first ferrocene-based antibiotics.

The dramatic bacterial resistance to antibiotics has motivated an urgent search for metallodrugs with potential antimicrobial activity. Acylation by ferrocene reagents of the commercially available β-lactam antibiotic 6-aminopenicillanic (6-APA), **23**, and 7-aminocephalosporanic acids (7-ACA) **24**, reported in Marr’ seminal work, produced the first ferrocene derivatives antibiotics **25** and **26**, although antibacterial activities were not improved compared with the original organic drugs [51] (Figure 8).

**Figure 9:**
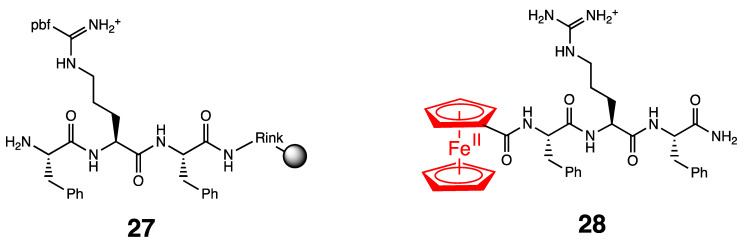
Chemical structure of peptide **27** and a ferrocene-containing derivative **28** that present antimicrobial activity.

The Metzler-Nolte group has developed very active bioorganometallics, including ferrocenyl-containing antimicrobial peptides (BOAMPs), which were synthesized by attaching the organometallic group to a preformed peptide before liberating the peptide from the solid phase of the peptide synthesizer [9,28,32,56]. For instance, the reaction of FcCO_2_H with the peptide **27** attached to the rink amide resin provided the ferrocenyl peptide **28**, after cleavage from the resin and deprotection of the side-chain protecting group, (Figure 9), with an enhanced antibacterial activity compared with that of the original peptide. The hydrophobicity of the ferrocene group was proposed to be responsible for the increased antibacterial activity of peptides, such as **27** in ferrocene-containing peptides, such as **28**, against strains of *Escherichia coli, Pseudomonas aeruginosa*, and *Staphylococcus aureus* [91]. Remarkably, the change from L to D of amino acids in the ferrocenyl-BOAMPs also provoked an increase in antibacterial activity, the most active ferrocenyl peptide derivatives sharing a C-terminal-Arg-D-Trp-NH_2_ moiety [92].

**Figure 10:**
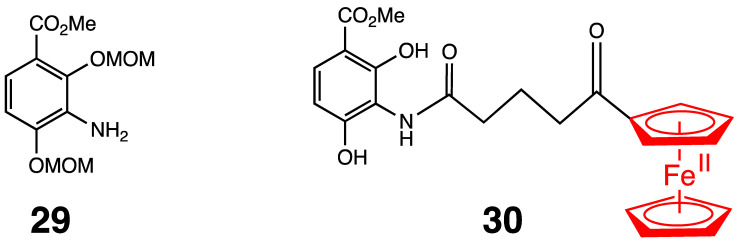
Chemical structures of platensimycin **29** and its ferrocenyl derivative **30**.

The introduction of ferrocene (or other organometallic groups) in the structure of an antibiotic molecule does not always result in an increase in antimicrobial activity. An example of an unfavorable situation was found with the platensimycin mimics **29**, for which the introduction of the ferrocenyl group in **30** resulted in low activity (128 mg/L against *Staphylococcus aureus* Mu50 VISA) [27,93] (Figure 10).

An account of the work by the Metzler-Nolte group on their efficient antibacterial organometallic peptide conjugates was published in 2017 [28], and a comprehensive review by the same group on organometallic-peptide bioconjugates and medicinal applications appeared in 2016 [94]. In addition, Sierra’s group published a mini-review on bioorganometallic derivatives of antibacterial drugs in 2017 [27]. In 2022, Hess proposed a selection of concepts to rationally design inorganic and organometallic antibiotics, highlighting their advantages by comparing them to classical drug discovery program [95]. The solid-phase synthesis and antibacterial evaluation of ferrocenyl water-soluble peptides is a subject of continued interest, and the groups of Gomez, Sierra, and Metzler-Nolte recently evaluated water-soluble cyclic metallocenyl, including ferrocenyl hexapeptide bioconjugates derived from the *homo*-sequence H-KKKKKK-NH_2_ by substitution of lysine (K) by tryptophan (W) and by orthogonal derivatization of the ε-*N*-amine group of lysine by a metallocene moiety. Insertion of two tryptophan residues and ferrocenyl moieties into linear *homo*-sequence peptides increased antibacterial activity with minimum inhibitory concentration (MIC) down to 5 μM [91].

Antimicrobial activity of RP-1 peptide (derived from the human chemokine CXCL4) conjugate with ferrocene group has been examined by Cilli and Graminha’s group with anti-amastigote activity against *Leishmania amazonensis* (IC_50_ = 0.25 mmol L^−1^). In comparison with amphotericin B, (IC_50_ = 0.63 mmol L^−1^), Fc-RP-1 was more active and showed a 2.5-fold higher selectivity index. The RP-1 peptide presented inter alia a MIC of 4.3 mmol L^−1^ against *Streptococcus agalactiae*, while Fc-RP-1 was 4 times more active (MIC = 0.96 mmol L^−1^), indicating that ferrocene improved the antimicrobial activity against Gram-positive bacteria [92].

**Figure 11:**
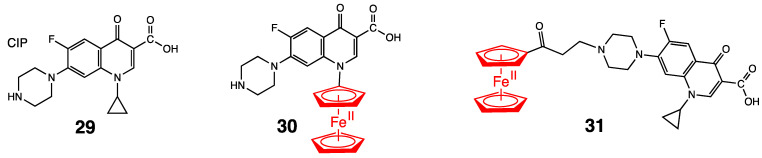
Chemical structures of ciprofloxacin and its ferrocenyl derivatives.

The antibiotic ciprofloxacin (**29**, Figure 11), a fluoroquinolone highly active against various microorganisms, is used for the treatment of respiratory and urinary tract infections and as a prophylactic for neutropenia and in veterinary medicine [96,97,98]. Biot’s group examined ferrocenyl-ciprofloxacin derivatives and found an antimalarial efficacy enhancement compared with ciprofloxacin, displaying IC_50_ values between 0.8–3.9 μm in chloroquine-resistant (W2) and chloroquine-sensitive (3D7) parasite strains. These complexes were active against both W2 and 3D7, and **30** (Figure 11) was the most active, with IC_50_ values of 1.0 and 0.8 μm against 3D7 and W2 [97]. A recent study by the group of Stazek and Kowalski elaborated that organometallic ciprofloxacin conjugates showed good antibacterial activity against a set of reference Gram-negative strains (*Escherichia coli* ATCC 25922, *E. coli* NCTC 8196, *Proteus vulgaris* ATCC 49990, and *Klebsiella pneumoniae* ATCC 13883) and Gram-positive strains (*Staphylococcus aureus* ATCC 6538, *Staphylococcus aureus* ATCC 29213, and *Staphylococcus epidermidis* ATCC 12228) and, additionally, against two clinical bone isolates of methicillin-resistant *Staphylococcus aureus* (MRSA) and *Staphylococcus Aureus subsp. Aureus Rosenbach* (ATCC 6538 CIPR). The ferrocenyl ciprofloxacin derivatives were active, in particular **31**, but less so than ciprofloxacin and some cyclopentadienyl manganese tricarbonyl ciprofloxacin derivatives [98].

**Figure 12:**
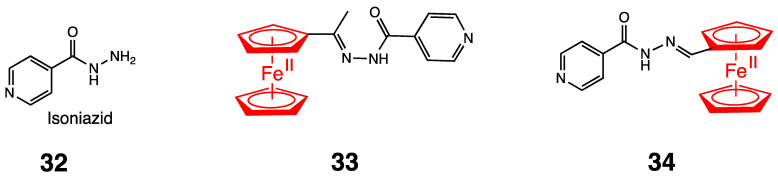
Chemical structure of the antibiotic isoniazid **32** and its ferrocenyl derivatives **33** and **34**.

**Figure 13:**
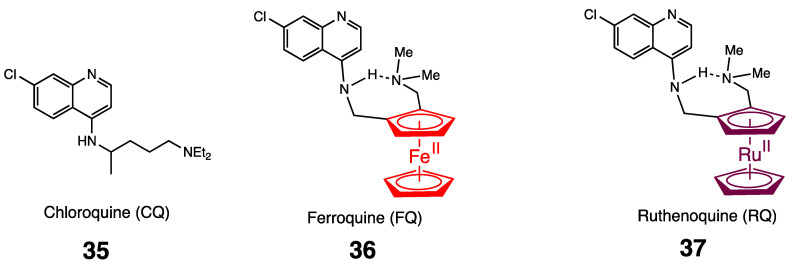
Chemical structures of Chloroquine, Ferroquine, and Ruthenoquine, presenting antimalarial activity.

Tirkey et al. reported the synthesis and activity test for bacterial strains *B. subtilis, E. coli, S. aureus, K. pneumoniae,* and *P. aeruginosa* of ferrocenyl hydrazone analogs, such as **33** of the antituberculosis (TB) drug isoniazid **32** (Figure 12). The superior antimicrobial activity of the ferrocenyl conjugate **33** (MIC values: 31.25–125 μg mL^−1^) compared with the organic drug **32** was assigned to the increased cell permeability and lipophilicity brought about by the ferrocenyl moiety, and possibly additionally to the π-electron delocalization and hindrance of metal binding sites in crucial enzymes [99]. Stringer’s group also reported ferrocenyl isoniazid conjugates, such as **34** (Figure 13), and tested them against malarial, trichomonal, and mycobacterial strains, with conjugates showing high antimycobacterial activity toward *Mycobacterium tuberculosis* (H37Rv strain) in the glycerol-based GAST-Fe growth medium (MIC_90_ or MIC_99_ < 1 μm), comparable to that of the free parent drug, **32**; however, **34** was not as active as **32** against *Mycobacterium tuberculosis* [100].

Trivedi’s group recently reported excellent antimicrobial activity of isatin (oxime)-triazole-thiazolidinedione ferrocene conjugates against some selected Gram-positive and Gram-negative strains, with the values comparable to the standard compounds Streptomycin and Flucanazole [101]. Kowalski;s group recently showed that ferrocenyl 7-aminocephalo-sporanic acid (7-ACA) antibiotic conjugate was more active against clinical *Staphylococcus aureus* isolates than the ampicillin reference. Moreover, no undesirable toxic effect in HeLa and L929 cells were observed at the concentrations at which they displayed strong antibacterial effects [102]. Kocak’s group recently screened the antimicrobial effect of ferrocene-boronic acid on *Pseudomonas aeruginosa* using proteomics and metabolomics approach and found inter alia that ferrocene-boronic acid affected various antimicrobial targets, such as ATP-dependent DNA helicase RecQ, transcription-repair-coupling factor, and primasome assembly protein PriA [103]. A review of organometallic antibacterial drugs and metal-based materials, including inter alia ferrocene conjugates with antibacterial activity, was recently published by Kowalski et al., and the conclusion was that combination of an organometallic moiety with an organic pharmacophore often resulted in circumventing drug resistance in bacteria, particularly via a bimodal mode of action. The authors also pointed out that the impaired uptake of some organometallic drug derivatives and their potential toxicity against mammalian cells hindered the full exploitation of the antibacterial activity at the clinical stage [104].

## 5. Antimalarial Ferrocenyl Conjugates

Given the resistance developed by malaria parasites, in particular by *Plasmodium falciparum*, to the main antimalarial drugs artemisinin (see **1**) and chloroquine (CQ) **35**, whose mechanism is inhibition of hemozoin biocrystallization, CQ and all the quinoline-based drugs have become ineffective against these strains. Thus, it was crucial to invent a novel strategy, in particular with metallodrugs, providing new antiparasite mechanisms. This challenge resulted in the 1994 discovery by the Biot-Brocard group of a ferrocenyl CQ derivative **36**, named ferroquine (FQ), in which a ferrocenyl group was incorporated into the alkyl chain binding the amino groups of CQ [105]. FQ was found to be active against both CQ-sensitive and CQ-resistant strains of *Plasmodium falciparum* (the most dangerous malaria species) in vivo and presently remains the most active among more than a hundred related metallodrugs of this series (Figure 13) [30,53,106]. FQ in association with artesunate passed in 2019 to phase IIb clinical trials. Ferroquine is a chiral molecule due to the metallocenyl chirality, but there is no significant difference in the activities of the pure enantiomers of FQ on parasites, and their cytotoxicities are similar. Thus, the racemate is used as an antimalarial drug [107].

In order to provide insight into how FQ acts against the *P. falciparum* parasite, Biot’s group has prepared and tested the ruthenocene analog, ruthenoquine (RQ) **37**, of FQ.

**Figure 14:**
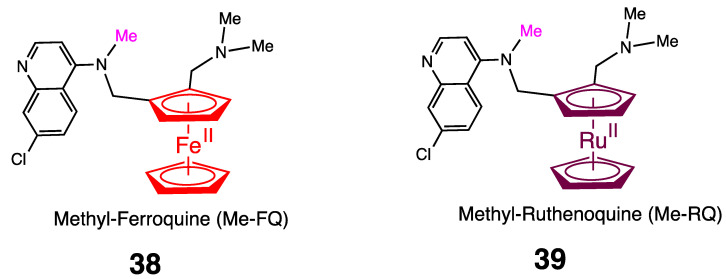
Chemical structures of methyl-ferroquine and methyl-ruthenoquine.

Although both FQ and RQ were active against malaria, FQ showed superiority over RQ in vivo in *P. vinckei*-infected mice, taking into account the lack of generation of OH. radicals from RQ, contrary to FQ. This way, FQ induced the breakdown of the parasite digestive vacuole membrane via OH. radicals, contrary to RQ. In addition, the hydrogen bonding in FQ (2.17 Å, broken in acidic media) and RQ between the two amine N atoms was shown to increase the hydrophobicity compared with CQ due to lowering the basicity that generated less protonated, thus more hypophilic species, which, together with the hydrophobicity brought about by the metallocenyl group, accelerated the penetration of the lipophilic digestive vacuole membrane faster than CQ does [108,109]. The authors also prepared and evaluated the methylated derivatives at the metallocenylmethylamine position, FQ-Me **38** and RQ-Me **39**, for which there is no possible hydrogen bonding between the two amine N atoms. Conversely, FQ-Me and RQ-Me, which do not involve this H-bonding between the two amino groups, have a toxicity mechanism related to CQ, contrary to FQ and RuQ (Figure 14).

The fact that FQ-Me and RQ-Me were less active than FQ against CQ-susceptible and CQ-resistant strains shows the crucial role of the H-bonding in the cytotoxicity. The ferrocenyl-lipid structure interaction allows FQ to escape the transport system involved in resistance, concentrating it where the FQ function is ideal for hemozoin formation inhibition. Like SQ, FQ also forms complexes with haematin (log K = 4.95), but it more strongly inhibits haematin crystallization into β-haematin than SQ.

**Figure 15:**
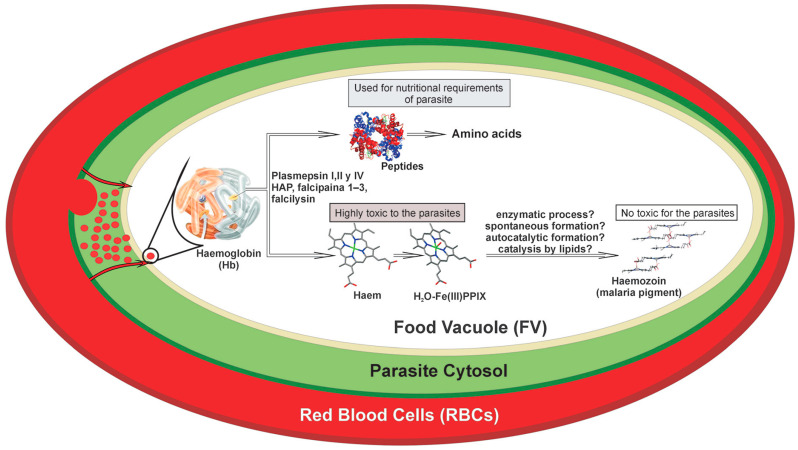
Process of hemoglobin degradation and heme detoxification by intraerythrocytic malaria parasite proposed by Biot et al. Reproduced with permission from Ref. [110]. Copyright 2010 American Chemical Society.

Production of OH. radicals was demonstrated by electron spin resonance (ESR) upon reaction of FQ with H_2_O_2_ in acidic media, but oxidation of RQ by H_2_O_2_ did not produce OH. radicals. The difference between ferrocene and ruthenocene in FQ resp. RQ is that ferrocene is reversibly oxidized according to a single-electron transfer, whereas ruthenocene oxidation proceeds with chemical irreversibility according to a two-electron transfer, because following electron transfer, the transient Ru(III) form dismutates to Ru(II) and unstable Ru(IV), and the pairwise reaction does not form OH. radicals. This difference in reactions between FQ and RQ with H_2_O_2_ matches the fact that FQ is more active than RQ in vivo. Hydroxyl radicals generated upon FQ treatment (contrary to CQ) provoke lesions in cell structures upon oxidation of unsaturated fatty acids in membranes, leading to vacuole membrane destruction and death of the parasite [108,109]. Reviews [109,110] by Biot et al. of their seminal and ongoing work have summarized the properties of the FQ drug families and proposed the overall FQ mechanism. In Figure 15, the mechanism of the intraerythrocytic malaria parasite proposed by Biot et al. is represented.

**Figure 16:**
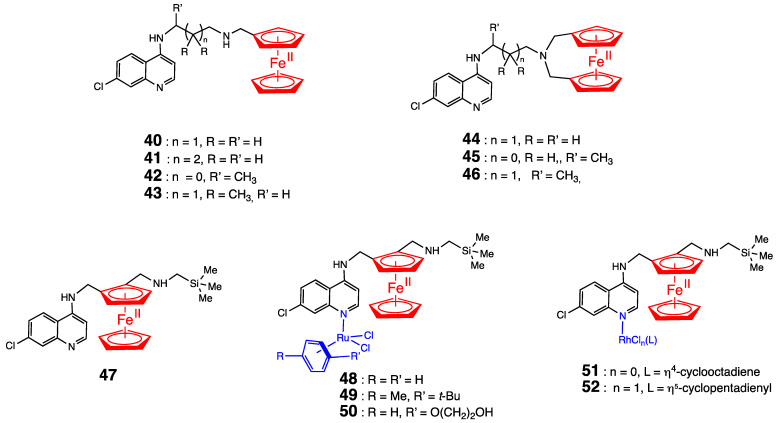
Efficient antimalarial mono-and bimetallic compounds derived from FQ.

Numerous FQ derivatives, including hydroxychloroquines, chloroquine-bridged ferrocenophane derivatives, trioxaferroquines, ferroquine dual conjugates, 4-*N*-substituted analogs, and other miscellaneous derivatives, have been synthesized and pharmacologically evaluated. Most of them were found to be superior to CQ, but only a few of them, shown in Figure 16, surpassed FQ itself in terms of antimalarial activity [110]. The trimethylsilyl derivatives **47**–**52** encountered some success because this group increased the FQ lipophilicity, and this series was extended to heterobimetallic derivatives, including FQ ruthenium and rhodium complexes **48**–**50** and **51**–**52**, respectively. In vitro, antiplasmodial activity of these compounds was established against the chloroquine-sensitive (NF54) and chloroquine-resistant (Dd2) strains of the malaria parasite *Plasmodium falciparum* [111].

**Figure 17:**
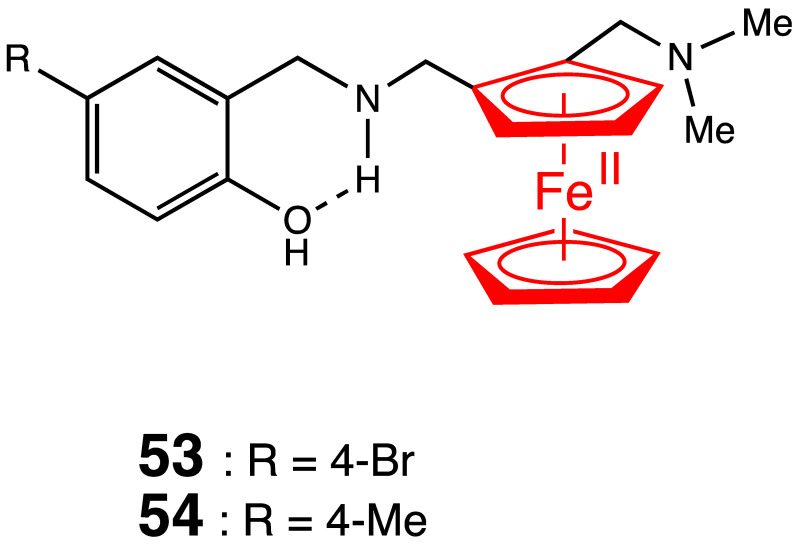
Potent antimalarial ferrocenyl derivatives containing the α-amino-*o*-cresol scaffold.

Parameters, including the H-bonding, such as in **36**, and the basic and hydrophilic/hydrophobic balance, have been compared and summarized. Briefly, **40**–**43** were more active than FQ against CQ-resistant K1 strains. The ferrocenophane derivatives **44**–**46** displayed higher activity than FQ against CQ-resistant K1 strains. The compounds **48**, **49**, **51**, and **52** exhibited better activity than FQ against the NF54 strain of *Plasmodium falciparum*, and the best FQ derivative **50** of this trimethylated series in the bottom of Figure 17 was 10-fold more active than FQ against the same parasitic strain [110,111]. The evaluation of systematic introduction of late transition-metal cation coordinating the amine groups of CQ and FQ has been reviewed very recently [112].

Antimalarial activities of other ferrocenyl derivatives have also been reported in addition to ferroquine. Biot’s group, together with the Medebielle’s and Bouillon’s groups and colleagues from several French laboratories, very recently reported the in vitro and physicochemical evaluation of ferrocenyl-containing hydroxy-lactam-derived tetramates that, interestingly, can exert potent antimalarial activities via a mechanism distinct from that demonstrated for ferroquine [113].

A family of ferrocenyl derivatives containing the antimalarial α-amino-*o*-cresol scaffold (represented in Figure 17 by the two most potent compounds **53** and **54**) showed antiplasmodial and selective antiparasitic activity with a phenolic OH group and rotatable C–NH bond responsible for biological activity and dual mode of action with hemozoin inhibition and DNA interaction. The compounds showed high selectivity toward the malarial 3D7 and Dd2 strains of the *Plasmodium falciparum* parasite, with no indication of cross-resistance and with low cytotoxicity against human umbilical vein endothelium cells (HUVEC). The preferential binding affinity of **53** for the plasmodial DNA over the mammalian DNA, together with hemozoin inhibitory affinity, substantiated the higher selectivity of the compounds for the *P. falciparum* strains. The aminocresol **54** was the most potent compound against the CQS 3D7 strain in the rich series with an IC_50_ value of 0.98 ± 0.10 μM [114].

Several other antimalarial ferrocene-containing drugs have recently been reported, including betulinic acid/betulin-derived dimers and hybrids [115] and amino-artemisinin-ferrocene derivatives [116,117,118]. Concerning the latter, artemisinin derivatives are known to promote cancer cell apoptosis, induce cell cycle arrest and autophagy, and inhibit cancer cell invasion and migration. Current artemisinin derivatives (including metal complexes) with potential anticancer activity have been very recently defined [119].

## 6. Antifungal Ferrocenyl Conjugates

**Figure 18:**
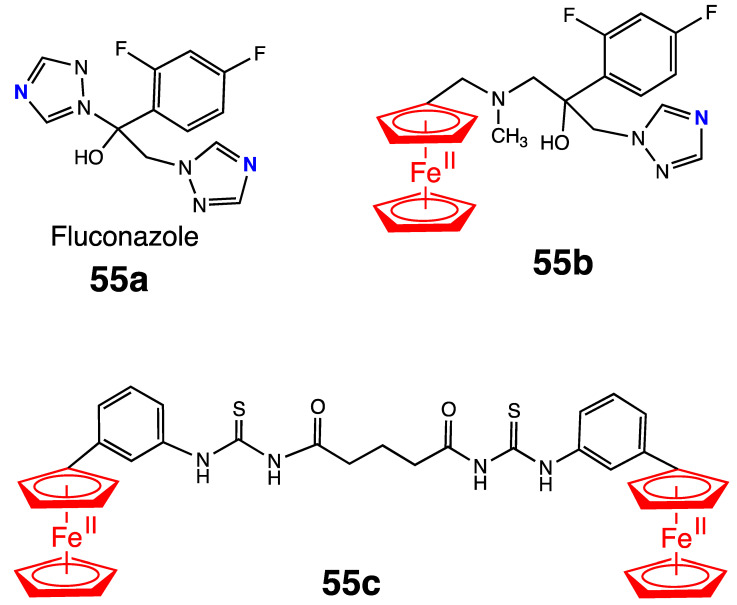
Representative antifungal compounds.

Fungal diseases are also causing global health problems, with treatments becoming ineffective due to the resistant strains of pathogens and high toxicity of drugs currently in use. The scarcity of efficient treatments for fungal diseases should motivate the research of novel drug candidates. In addition to the seminal finding by the Biot-Brocard group of the antifungal activity of a ferrocene derivative of fluconazole **55a** (Figure 18) [54], one of the most common antifungal drugs, active against a large number of mycoses, a recent in vivo study by Gasser’s group reported the synthesis and activity of four new fluconazole derivatives. The authors focused on azoles, the mainstay of antifungal drugs, their activity involving their binding via the triazole nitrogen atom marked in blue in **55a** and **55b** to the heme iron atom of the fungal lanosterol 14α-demethylase target enzyme (from computing binding geometry data). The activity of **55b** was shown on clinical isolates, with antimycotic potency up to 400 times higher than fluconazole, and activity toward azole-resistant strains was demonstrated (replacement of the methyl group of **55b** by H or larger groups showed that the methyl group provided the best results). In vivo studies in a mice model of *Candida* infections showed that **55b** not only reduced the fungal growth and dissemination but also improved immunopathology [120].

**Figure 19:**
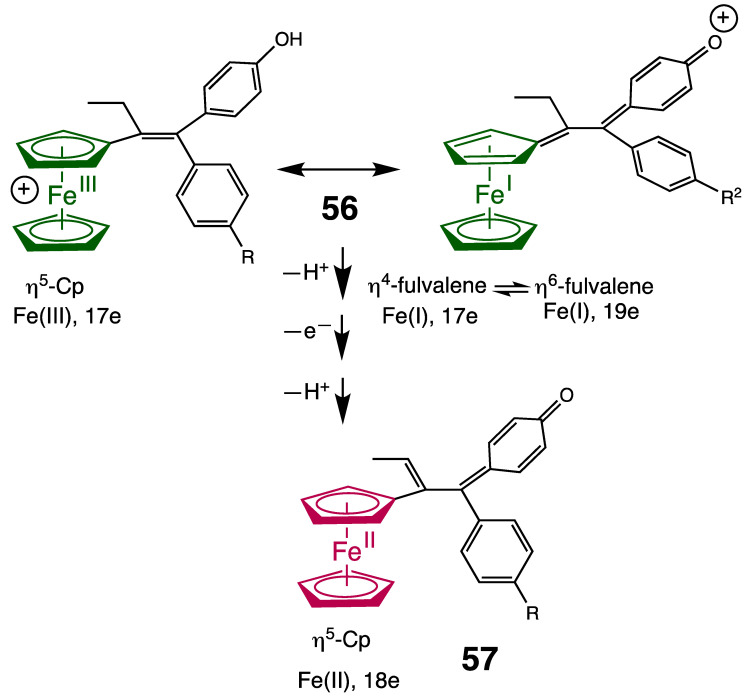
Proposed structure **56** resulting from the single-electron oxidation of ferrocifen **11**. Upon double proton-coupled electron transfer, **11** was oxidized to **57** [7].

Ferrocenyl drug conjugates showing antifungal activity also show other biological properties, such as antibacterial, antimalarial, and/or anticancer properties. Along this line, additional Schiff-based ligands and their Zn and Cu complexes were found in antibacterial and antifungal agents in vitro, with antifungal properties enhanced by the complexation of the Schiff-based ligand to these metals [62]. Kaliappa’s group synthesized ferrocenyl conjugates containing nonsymmetrical tetradentate Schiff-base ligands that were shown to possess antimicrobial activity against four bacterial and two fungal stains [121]. Recently, Cilli et al. developed new strategies for ferrocene-containing antimicrobial RP-1 peptides, synthesized through solid-phase peptide synthesis, with antifungal and antiplasmodial properties with low toxicity in the U87 and HepG2 cell lines [122]. Badshah’s group reported new symmetrical ferrocene-based bisthiourea analogs and examined their antibacterial, antifungal, antioxidant, and antidiabetic activities in vitro. In particular, antifungal studies conducted using a disc diffusion method against *Mucor* species (FCBP 0300), *Aspergillus niger* (FCBP 0198), *Aspergillus flavus* (FCBP 0064), and *Fusarium solani* (FCBP 0291) showed activity against all fungal species with maximum activity against *Aspergillus flavus* and *Aspergillus niger*. The most active derivative in the series was **55c** (Figure 19) [123]. Ferrocenyl-containing chalcone derivatives have also been recently shown by various research groups to possess inter alia antifungal properties [82,83,124].

## 7. The Fenton Reaction with Fe^2+^ and Ferrocene Derivatives

The Fenton reaction, discovered by Fenton at the end of the 19th century [125], involves the reaction of a ferrous (Fe^2+^) salt (in water, Fe^2+^ is in the form of [Fe(H_2_O)_6_]^2+^.) [126] with H_2_O_2_ (Equation (1)), followed by the slower reaction of the ferric (Fe^3+^) salt with H_2_O_2_ (amphoteric role of H_2_O_2_, Equation (2)). This results in the consumption of Fe^2+^, via the intermediate [Fe(III)OOH]^2+^, formed according to Equation (3) in a very acidic medium. External energy fields, including light, heat, ultrasound, electric, or magnetic fields, can also promote Fenton reactions. In the photo-Fenton mechanism, the intermediate [Fe(III)OOH]^2+^ is photolyzed to HO_2_. and Fe^2+^ (Equation (4)) [127] but, with ferrocene, the Cp ligands are not lost, which inhibits this process. On the other hand, H_2_O_2_ can be photolyzed to OH. (Equation (5)). The intermediacy of the OH. radicals at pH < 3 was shown much later, in 1934, by Haber and Weiss [128], and observed by EPR in 1979 [129]. In the following equations, the H_2_O ligands are omitted.

(1) $${\mathrm{Fe}}^{2+}+H_{2}O_{2}\to {\mathrm{Fe}}^{3+}+{\mathrm{OH}}^{-}+{\mathrm{OH}}^{•}$$

(2) $${\mathrm{Fe}}^{3+}+H_{2}O_{2}\to {\mathrm{Fe}}^{2+}+H^{+}+{\mathrm{HO}}_{2}•$$

(3) $${\mathrm{Fe}}^{3+}+H_{2}O_{2}\to {[\mathrm{Fe}(\mathrm{III})\mathrm{OOH}]}^{2+}+H^{+}$$

(4) $${[\mathrm{Fe}(\mathrm{III})\mathrm{OOH}]}^{2+}+hv\to {\mathrm{Fe}}^{2+}+{\mathrm{HO}}_{2}•$$

(5) $$H_{2}O_{2}+hv\to 2{\mathrm{OH}}^{•}$$

The pH is crucial to the Fenton reaction, for instance, because of the hydrolysis of Fe^2+^ to Fe(OH)_n_^2−n^ species (*n* = 1–4) if the pH is not acidic (including, for *n* = 2, precipitation of ferrous hydroxide) [130].

Thus, in an acidic medium, Equation (1) becomes Equation (6):

(6) $${\mathrm{Fe}}^{2+}+H_{2}O_{2}+H^{+}\to {\mathrm{Fe}}^{3+}+H_{2}O+{\mathrm{OH}}^{•}$$

For mammalian cells, the intracellular pH value varies from 4.7 in lysosome to 8.0 in mitochondria, and cancer cells are more acidic than healthy cells. The efficiency of the Fenton reaction is optimal in the pH range between 2 and 4. In consequence, the mildly acidic tumor microenvironment is not ideal for the Fenton reaction. Thus, reducing the pH in tumor tissues must be fulfilled by chemodynamic therapy (CDT). Likewise, endogenous H_2_O_2_ is insufficient to induce an efficient Fenton reaction. An excellent method to decrease the pH and increase the H_2_O_2_ level in the tumor microenvironment (TME) is to include glucose oxidase (GOx) in the CDT cargo because GOx catalyzes, using the co-factor Flavine Adenine Dinucleotide (FAD), the oxidation of β-D-glucose to H_2_O_2_ and D-glucoco-1,5-lactone that further hydrolyzes to gluconic acid (vide infra).

The hydroxyl radical OH. produced in the Fenton reaction is an extremely high-energy species [131], its potential *E*° (OH·/H_2_O) being as high as 2.81 V vs. normal hydrogen electrode (NHE). In the presence of excess H_2_O_2_, the radical OH. is oxidized, yielding the other radical HO_2_· (Equation (5)), the protonated form of superoxide radical anion O_2_·^−^ (Equation (7)), with a *pK_a_* value of 4.8 for HO_2_.

(7) $$H_{2}O_{2}+{\mathrm{OH}}^{•}\to H_{2}O+{\mathrm{HO}}_{2}•$$

The superoxide radical anion, O_2_·^−^ [132], and its protonated form HO_2_. are also formed, depending on the pH, but the basic and nucleophilic properties of O_2_·^−^ are highly dependent on whether the medium is aqueous or nonaqueous [133]. HO_2_ is highly unstable in water and dismutates to H_2_O_2_ and O_2_ (Equation (8)). In a hydrophobic medium, the basic and nucleophilic reactions are favored, and dismutation occurs under the influence of a catalyst, particularly a superoxide dismutase (SOD) enzyme [132].

(8) $${2\mathrm{HO}}_{2}•\to H_{2}O_{2}{+O}_{2}•$$

The radical HO_2_· also reacts with H_2_O_2_ to yield OH·, but this reaction is too slow to significantly contribute to the production of the radical OH. (Equation (9))

(9) $$H_{2}O_{2}+{\mathrm{HO}}_{2}•\to H_{2}O+{\mathrm{OH}}^{•}{+O}_{2}•$$

The radicals OH·, HO_2_·, and O_2_·^−^ belong, together with peroxide (ROOR), singlet oxygen (^1^O_2_), and nitric oxide (NO.), to the so-called reactive oxygen species (ROS) that are byproducts of the normal biological oxygen metabolism, present at low and stationary levels in normal cells, and known for their role in cell signaling and homeostasis; they are also known for causing irreversible damage to intracellular DNA [134]. However, the OH. radical is by far the most reactive among the ROS.

**Scheme 2:**
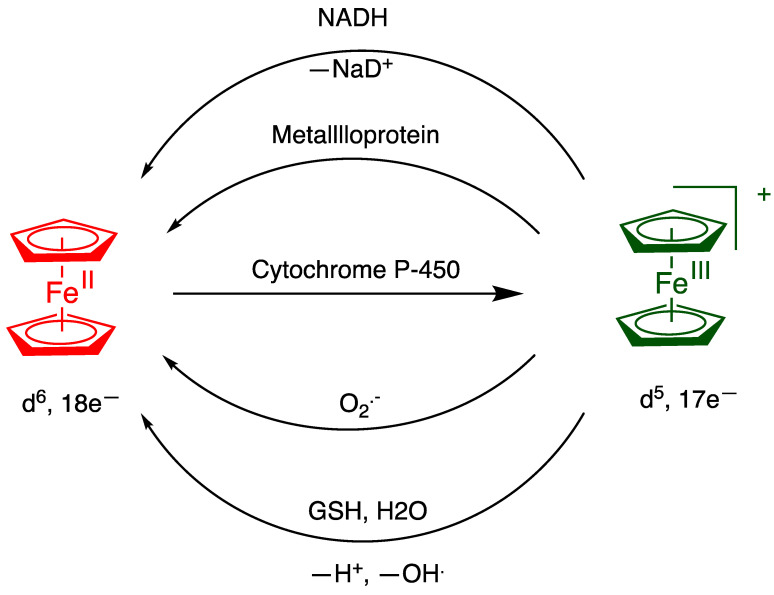
Ferrocene/Ferricenium redox system: redox exchange by biological reagents. Adapted with permission from Ref. [5]. Copyright 2005 Elsevier.

Since both ferricenium and ferrocene derivatives have long been known for their anticancer and antimicrobial activities, it became essential that the redox stability of both redox forms in biological media (Scheme 2) (i.e., the electron-reservoir property of ferrocene) [135] was a key aspect in their pharmacological properties, as noted early in [5,136]. The long-term degradation of ferricenium salts in vivo is still in question. The lipophilic neutral form allows for transferring the derivative through biological membranes, whereas the cationic oxidized form provides solubility in hydrophilic media.

The ability of the oxidized form of ferrocene, ferricenium, to produce cytotoxic OH. radicals in the Fenton reaction was demonstrated by Hiroshi and Masahiro [36] in 1997 and confirmed by Osella et al. [37] in 2000 by electron paramagnetic resonance (EPR) characterization. Major differences between the oxidation of the two Fe(II) complexes [Fe(H_2_O)_6_]^2+^ and ferrocene (FeCp_2_) are the solubilities, the coordination, and the redox potential *E*°(Fe^III/II^). The salts [Fe(H_2_O)_6_]^2+^(X^−^)_2_ are mostly water-soluble, whereas ferrocene is soluble in nonpolar organic solvents and insoluble in water. Another key difference between this classic Fenton reaction of Equations (1)–(3) with Fe^2+^ and the Fenton reaction with ferrocene or ferrocenyl derivatives is that with the latter, Fe(II) in ferrocene is coordinatively saturated (FeCp_2_ is a robust 18-electron complex), and thus the radicals OH· and HO_2_· cannot bind to the Fe center. This is in contrast with hydrated Fe^2+^, in which the H_2_O ligands are easily deprotonated or displaced by hydroxy anions or radicals.

**Scheme 3:**
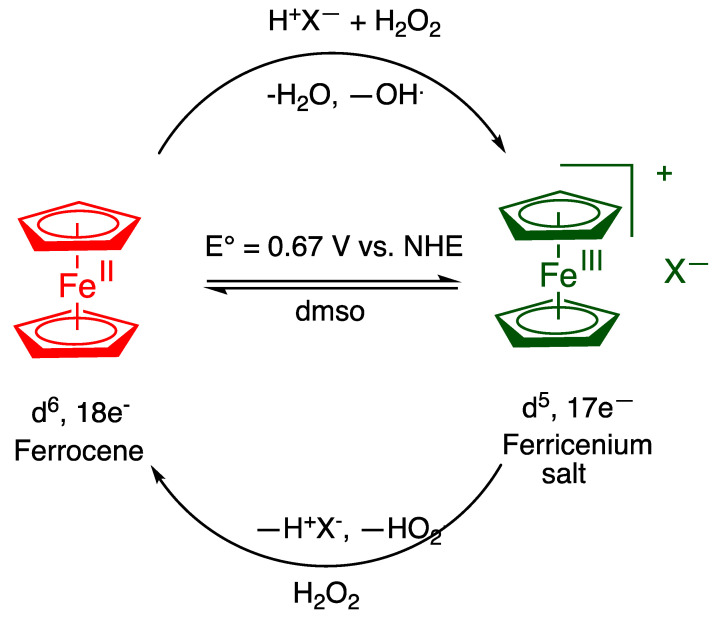
Fenton reactions involving ferrocene and ferricenium salts.

The redox potential determines the rate of Fe(II) oxidation; [Fe(H_2_O)_6_]^2+^ is oxidized at 0.77 V vs. NHE in water, whereas ferrocene is oxidized at 0.67 V vs. NHE in DMSO, which means that ferrocene is oxidized slightly more easily than [Fe(H_2_O)_6_]^2+^, although comparison between reactions in water and organic solvents is difficult (Scheme 3).

Another aspect is that ring substituents influence the redox potential of the ferrocenyl group. In particular, when the ferrocenyl moiety bears one or two carbonyl groups on the rings, oxidation becomes more difficult by 0.3 V or 0.6 V, respectively, compared with that of ferrocene [137,138]. Lacina’s group reported that the cytotoxicity of ferrocene derivatives and ROS-activated prodrugs based on ferrocenyliminoboronates depended on their redox potential, aminoferrocene with its lowest redox potential exhibiting the highest cytotoxicity [139].

**Figure 20:**
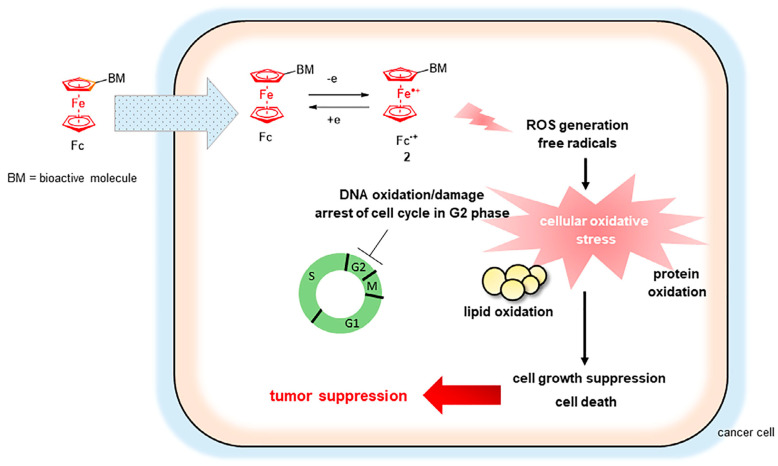
Mechanism of oxidative stress leading to cell death provoked by Fenton reaction with a ferrocenyl conjugate. Reproduced with permission from Ref. [26]. Copyright 2019 Elsevier.

Many ferrocenyl-containing drugs bear a group conjugated (styrenyl, triazole, chalcone, etc.) with the Cp ligand in the Fe(II) and Fe(III) forms, which stabilizes them and lowers the oxidation potential, facilitating the production of hydroxyl radicals. For instance, concerning the ferroxifen of type **11** [7], one can propose that, upon single-electron oxidation, the mono-oxidized species **56** is stabilized by the dual resonant forms (Figure 20), including the fulvalene structure (isolobal to the redox catalysts CpFe(I)arene) [140,141], which explains the proton-coupled electron transfer [142] viewing the strong acidity of the oxenium form (Figure 19).

Ultimately, the quinone methide complex **57**, spontaneously obtained at the same potential after two proton-coupled electron transfer sequences, is also more easily oxidized than ferrocene because of the conjugated organic fragment. Complex **57** is thus cytotoxic, responsible for hydroxyl radical generation, and, as a metabolite following treatment with ferrocifen, has been found to likely cause cell death upon interaction with DNA, proteins, and glutathione. Ferrocifen quinone methides are primary metabolites undergoing Michael additions with thiols or selenols of key proteins, which generates a disruption of cell metabolism [143].

This condition is not determining, however; for instance, the ferrocenophane (ansa-ferrocenes) derivatives **12** of the ferrocifen family inhibiting conjugation with the substituent show excellent and sometimes better activity than fully conjugated ferrocifens **11** [7].

The ferrocifens produce ROS at the 1 mM incubation concentration after 10 min incubation, unlike tamoxifen and hydroxytamoxifen. The addition of the antioxidants *N*-acetyl cysteine or vitamin E leads to the loss of antiproliferative effect of ferrocifens, which demonstrates that the toxicity of these ferrocenyl drugs is due to their production of ROS. However, the formation of ROS is not connected to the presence of a phenol group, and the quantity of ROS produced is also independent of the IC_50_ values.

In the mechanism of the oxidation of a series of ferrocene derivatives with hydrogen peroxide, Fomin and Zaitseva observed a shift of λ_max_ of the absorption band of the ferricenium cation toward long wavelengths and its broadening during the reaction, which was assigned to the formation of a {ferricenium cation + OH.} radical pair [144]. Clearly, organoiron radicals play a key role in reactivity [145], especially with such a high-energy species as OH.

ROS at a low level are cellular signaling messengers via modification of protein structure in healthy cells, but high ROS levels disrupt normal cellular processes via nonspecific attack of proteins, lipids, and DNA, thiol groups of proteins and glutathione being privileged targets. The production of ROS by cancer cells is due to increased metabolic rate, mitochondria dysfunction, oncogene expression, increased peroxisome activities, and elevated cell signaling. The Fenton reactions providing excess ROS provoke DNA oxidative damage and cell cycle arrest (Figure 20) [146].

Likewise, with ferroquine, the lipophilic ferrocenyl group produces ROS within the digestive vacuole, provoking membrane damage, due to the specific acidic and oxidative digestive vacuole conditions. The hydroxyl radicals produced upon ferrocenyl group oxidation by H_2_O_2_ attack unsaturated fatty acids in the phospholipid membrane [110].

Finally, note that the classic and efficient method of ROS generation, tumor irradiation with X-rays, is not without danger for normal tissues on the X-ray path [147,148]. On the chemotherapeutic side, doxorubicin (DOX) is believed to fight tumors through the generation of DNA damage by the DOX-induced direct H_2_O_2_ generation [149]. In addition to Fe(II), MnO_2_-coated mesoporous silica was also reported to yield hydroxyl radicals via glutathione depletion [150]. In general, other chemodynamic therapy (CDT) agents, including Mn^2+^ (vide supra), Ti^3+^, Cr^3+^, Ru^2+^, Cu^2+^, Co^2+^, Ce^3+^, persulfate, and peroxymonosulfate, have been shown to possibly replace Fe^2+^ toward hydroxyl radical production [151]. Along this line, various nanomaterials have also been proposed [152].

## 8. Drug Delivery Using Ferrocene-Containing Nanomaterials

### 8.1. Introduction and Early Studies

Recent giant progress in nanomaterials design has allowed for their optimization for drug delivery [38]. Such nanomaterials include organic materials, such as polymers [153], micelles [154], liposomes [155], dendrimers [155,156,157,158,159], cyclodextrin [160], and inorganic materials, such graphene [161], quantum dots [162], gold nanoparticles [163,164,165], coordination polymers, and metal–organic frameworks (MOFs) [166], and other nanomaterials [41] that can be involved in the generation of ROS [167].

**Figure 21:**
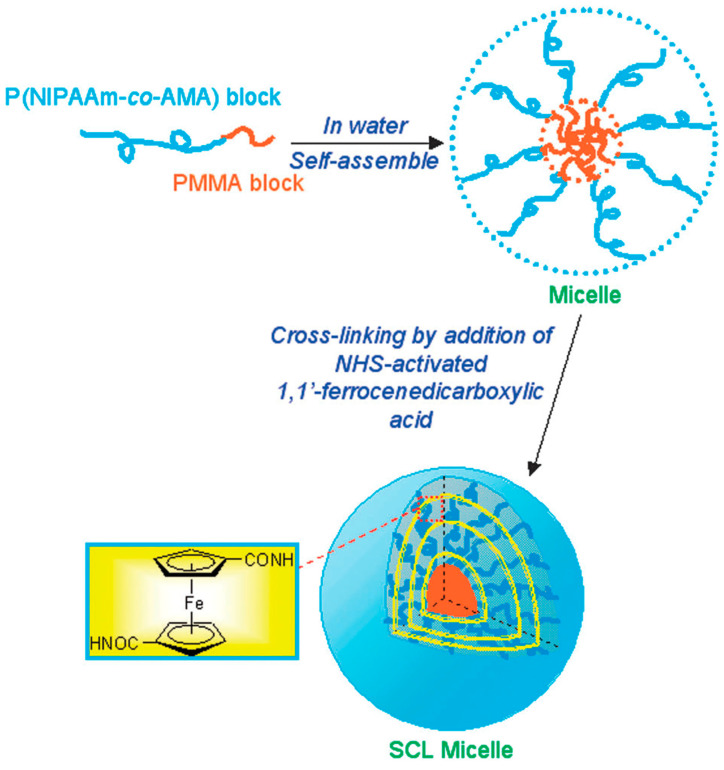
Formation of a shell cross-linked thermoresponsive micelle containing a 1,1′-bis-amidoferrocene. Reproduced with permission from Ref. [168]. Copyright 2010 American Chemical Society.

Most of these nanomaterials have been loaded with ferrocenyl groups to increase water-solubility and blood circulation lifetime of small molecules toward Fenton generation of hydroxyl radicals for a therapeutic purpose. This field was pioneered by Neuse with ferrocenyl-containing water-soluble polyaspartamide copolymers probed against HeLa cells and the COLO 320 DM with IC_50_ values in the range of 3.6–36 mM for the best derivatives. In the latter, amine groups provide cationic ammonium for cell entry [5]. The groups of Jaouen [49] and Romao [50] investigated the inclusion of ferrocifens and 1,2-disubstituted ferrocenes, respectively, into b-CD and found an increase in drug solubility without cytotoxicity decrease. Moreover, with heptakis-2,3,6-tri-O-methyl-b-CD (TRIMEB) or 2-hydroxypropyl-b-CD (HPbCD), cytotoxic activity increased by 2.5 times. The shell cross-linked hybrid 1,1′-bis-amidoferrocene-containing self-assembly of poly(N-isopropylacrylamide-co-aminoethyl methacrylate)-b-polymethyl methacrylate (P(NIPAAm-co-AMA)-b-PMMA) polymeric micelle showing reversible dispersion/aggregation in response to temperature cycles exhibited significant cytotoxicity against HeLa cells with a IC_50_ around 0.2 g/L (Figure 21) [168].

The Passirani-Benoit group studied local delivery of Jaouen’s ferrocifenol **11a** (Figure 4, Fc-diOH) on a 9L glioma model according to two encapsulation strategies: lipid nanocapsules (LNC) and swollen micelles. At the micellar interface, PEG prevented interaction with the cytoplasm membrane, leading to low micelle cell uptake and no biological activity. On the other hand, Fc-diOH (**11a**) cytostatic activity was preserved after encapsulation in LNC, being very effective on 9L-glioma cells (IC_50_: 0.6 mM). Rat treatment with Fc-diOH-LNC dramatically reduced the tumor mass and glioma volume. Moreover, Fc-diOH-loaded LNCs showed low toxicity levels for healthy cells [49]. Further study by these authors of Fc-diOH in LNC was conducted in combination with external radiotherapy against intracranial 9L glioma. Combining cerebral irradiation (three irradiation of 6 Gy doses, total dose: 18 Gy) with convection-enhanced delivery of Fc-diOH-LNCs led to long-term survivors compared with Fc-diOH-LNCs-treated animals (*p* < 0.0001) and the group treated with blank LNCs + radiotherapy (*p* = 0.0079). Rats survived over 100 days, indicating total eradication of the tumor. This study was the first demonstration of a synergy between these organometallic compounds and external beam radiotherapy [169].

### 8.2. Polymers, Micelles, and Liposomes

Bio-inspired ferrocene-containing polymers are an emerging field with multiple applications in pharmacotherapy [170,171]. Supramolecular polymers (SP) have been designed to build up nanosized delivery platforms for tumor chemotherapy [172,173,174,175]. Supramolecular nanoparticles were assembled via a simple one-step self-assembly process using platinum (IV) complex-modified b-CD-ferrocene conjugates and rapidly dissociated upon exposure to endogenous H_2_O_2_ in the tumor and release. This way, they released in situ hydroxyl radicals and platinum (IV) prodrug that was reduced to cisplatin, promoting the generation of H_2_O_2_ in the tumor tissue [172]. There are a few examples reported of SP construct using Fenton reagents as the guests for cancer imaging and treatment [176,177]. Ferrocene-containing amphiphilic block copolymers PEG-b-PMAEFc, synthesized by atom transfer radical polymerization (ATRP) of 2-(methacryloyloxy) ethyl ferrocene-carboxylate (MAEFc) using a PEG-based macro-ATRP agent, self-assembled into various nanostructures in aqueous solution and were treated with H_2_O_2_ to trigger the release of encapsulated cargo (rhodamine B) from b-CD in the polymeric nanocarriers [178]. A hydrogel with ferrocene groups in a polymer network immersed in water-soluble pillar[6]arene (WP6) aqueous solution was swollen, with approximately 11-fold increase in weight compared with that in pure water, due to the formation of the inclusion complexes between WP6 and ferrocene groups in the hydrogel. It exhibited good responsiveness to temperature, pH, redox, and competitive guests by tuning the dissociation/formation of WP6 ferrocene inclusion complexes [179].

These supramolecular properties of ferrocene-containing polymers have been applied in a few recent cases to the Fenton-like generation of hydroxyl radicals, as follows. Assemblies in the range of 100 nm accumulate around the tumor via the enhanced permeability and retention (EPR) effect and, upon exposure to the TME, ferrocene moieties herein undergo a Fenton-like reaction resulting in size shrinkage to 10 nm, thereby improving the tumor penetration ability of the assembly. These nucleic acid assemblies release hydroxyl radicals into the TME for efficient cancer treatment [180]. In an intracellular acidic environment, a pH/ROS dual-responsive supramolecular polypeptide nanoprodrug, PFW-DOX/GOD, consisting of a pH-responsive ferrocene/pillar[5]arene-containing polypeptide, a ROS-responsive polyprodrug, and encapsulated glucose oxidase (GOx), show pH-triggered disassembly. Simultaneously, the released GOx catalyzes intratumoral glucose into H_2_O_2_ that is further converted into toxic hydroxyl radicals from the ferrocenyl unit [181]. A biodegradable covalent organic porous polymer (COP), denoted as Fc-Ma, composed of ferrocene and D-mannitol (Ma) with pH-responsive acetal bonds, catalyzes the conversion of cellular H_2_O_2_ to hydroxyl radicals through a Fenton-like reaction, as an artificial enzyme. It also shows doxorubicin hydrochloride (DOX) drug loading capacity of 64.4 resulting in controlled drug delivery with selective release in acidic media [182]. Green-fluorescent, cancer-cell-targeted, and cationic carbon dots (HP-CDs) were specifically internalized with cancer cells, due to the recognition of surface hyaluronic acid with highly-expressed CD44 receptor. Ferrocenylseleno-dopamine (FcDA) was assembled on the surface of these HP-CDs yielding CDs@FcDA nanoprobe. Upon the cellular-specific uptake, the ferrocene moiety of the nanoprobe was oxidized by intracellular H_2_O_2_ in a typical Fenton-like reaction producing a toxic hydroxyl radical that provoked cancer cell apoptosis [183].

**Figure 22:**
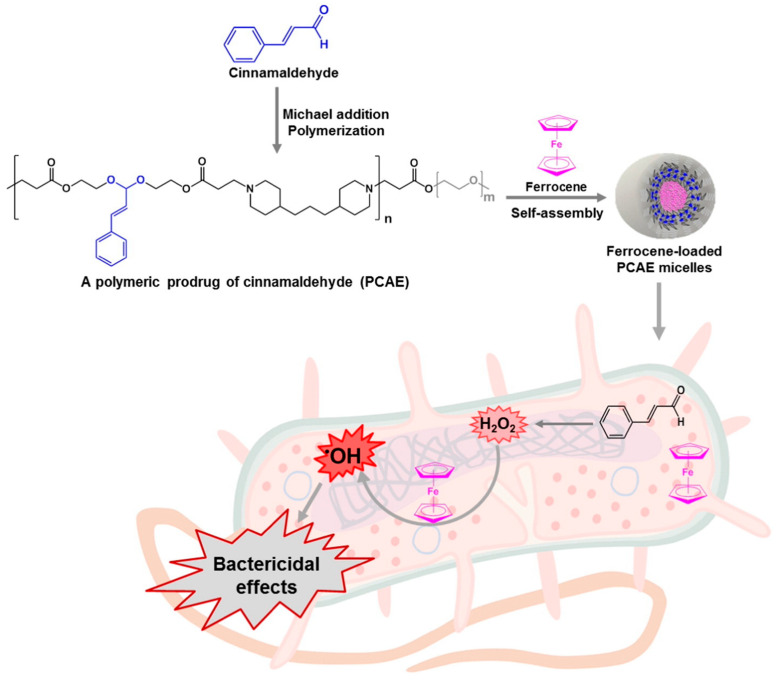
Mechanism of the bactericidal effect of produced hydroxyl radical subsequent to ferrocene@micelle encapsulation, in vivo delivery, and Fenton reaction. Reproduced with permission from Ref. [184]. Copyright 2016 Elsevier.

Amphiphilic PCAE incorporating H_2_O_2_-generating polymer, consisting of poly [(3-phenylprop-2-ene-1,1-diyl)bis(oxy)bis(ethane-2,1-diyl)diacrylate]-*co*-4,4′(trimethylene dipiperidine)-*co*-poly (ethylene glycol), termed PCAE, in which cinnamaldehyde was covalently incorporated in its backbone through an acid-cleavable linkage and self-assembled into stable micelles encapsulating ferrocene in their hydrophobic core, produced hydroxyl radicals killing *Escherichia coli* and *Pseudomonas aeruginosa* through membrane damage. Intraperitoneally injected ferrocene-loaded PCAE micelles reduced lung damage and increased the survival rate of mice infected with drug-resistant *P. aeruginosa* (Figure 22) [184].

**Figure 23:**
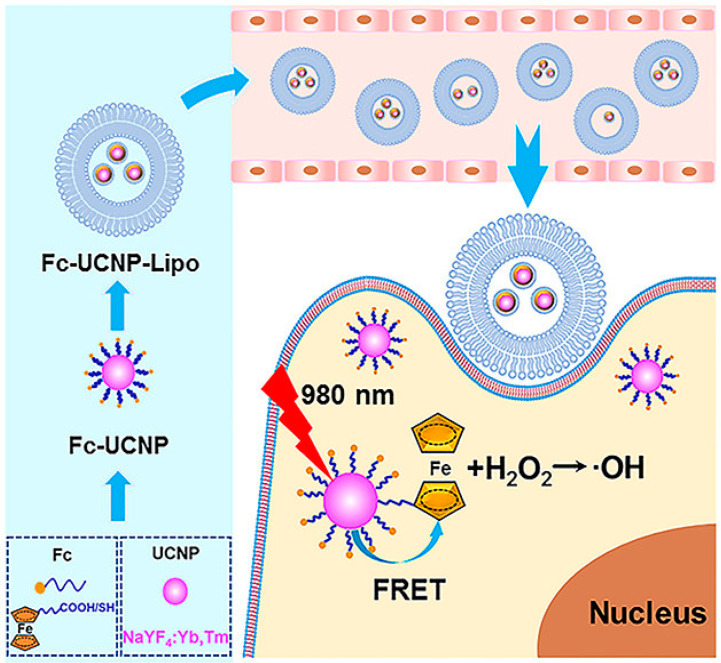
Fenton reaction from FRET between NIR-responsive lanthanide-doped upconversion nanoparticles and ferrocenyl derivative by functionalizing NaYF4:Yb,Tm NPs with ferrocene by encapsulating the NPs inside liposomes for delivery. Reproduced with permission from Ref. [187]. Copyright 2020 American Chemical Society.

A micellar system (DOX/FCH), composed of ferrocene and hyaluronic acid (HA), delivered doxorubicin (DOX) in about 50% in 2 h due to disulfide bonds depolymerization in reducing environment simulating tumor intracellular environment, thanks to high affinity between HA and high-expressed CD44 receptors in human cervical carcinoma (HeLa) cells. Hydroxyl radicals produced by the Fenton reaction were detected both extracellularly and intracellularly, showing synergistic effects of chemodynamic therapy of ferrocene and chemotherapy of DOX [185]. Along this line, an amphiphilic copolymer poly(ethylene glycol) (PEG) ferrocenyl monomethyl ether formed stable micelles in water and loading DOX reacted with H_2_O_2_, which led to hydroxyl radicals via the Fenton reaction, depleting glutathione (GSH) and suppressing the antioxidant capacity of tumor cells. The synergistic effects of DOX and ferrocene optimized the therapeutic impact [186]. Forster resonance energy transfer (FRET) between near-infrared (NIR)-responsive lanthanide-doped upconversion nanoparticles (UCNPs) and ferrocenyl compounds was organized by functionalizing NaYF4:Yb,Tm NPs with ferrocene via the surface coordination followed by encapsulating the NPs inside liposomes for delivery. In vitro and in vivo studies of hydroxyl radical generation by Fc-NPs-liposome showed preferential accumulation in a tumor site followed by enhanced uptake of cancer cells (Figure 23) [187].

### 8.3. Cyclodextrins and Pillar[6]arenes

Since Breslow’ seminal work [188], CDs have been shown to be hosts of choice for the use of ferrocenyl derivatives as guests in biomedicine [180,189,190,191]. pH-responsive and ferrocene-containing block copolymers, including b-CD, were synthesized to realize pH-stable and multiresponsive glucose oxidase-loaded polymersomes as a permeable membrane providing chemoenzymatic cascade reactions [189]. A polymeric NP that selectively improved H_2_O_2_ levels in tumor tissue and generated active hydroxyl radicals by the Fenton reaction was proposed. It used a diblock copolymer containing PEG and poly(glutamic acid) modified by b-CD coassembled in aqueous solution with block copolymer, ferrocenecarboxylic acid hexadecyl ester (DFc), and ascorbyl palmitate (PA) yielding a core shell micelle by the complexation of the ferrocenyl group inside the b-CD. Upon intravenous injection, this nanomaterial achieved significant accumulation in tumor tissue where, subsequently, the Fenton reaction between H_2_O_2_ and Fc groups produced hydroxyl radicals efficiently killing cancer cells and suppressing tumor growth [190]. Dual-responsive supramolecular self-assembled NPs based on polymerized methacrylic acid and polymerized poly(ethylene glycol) dimethyl acrylate-modified 6-CD with Fc-connected (S) (+)-camptothecin was taken up by tumor cells owing to their reversible negative-to-positive charge transition capacity at acidic pH. This supramolecular nanomaterial increased ROS generation, decreased GSH to amplify oxidative stress, improving the therapeutic effect and displaying good drug delivery capabilities to tumor cells or tissues, and inhibited cancer cell proliferation in both the cells and tissues [191].

**Figure 24:**
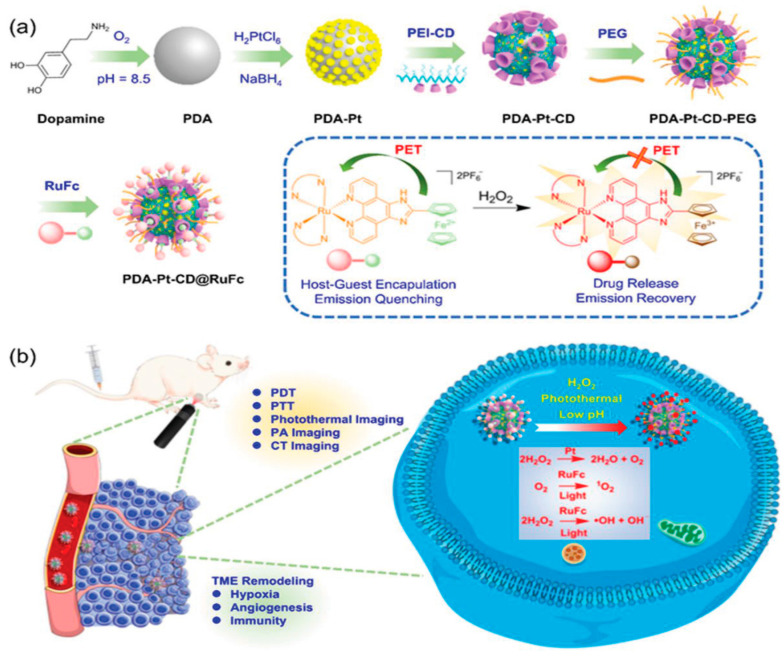
(**a**) Construction of polydopamine (PDA)-Pt-CD@RuFc NPs. Drug release and fluorescence recovery due to oxidation of the ferrocene group in RuFc by H_2_O_2_ are shown in the frame. (**b**) Proposed action mechanisms of PDA-Pt-CD@RuFc NPs. Reproduced with permission from Ref. [192]. Copyright 2020 Wiley.

A nanoplatform polydopamine(PDA)-Pt-CD@RuFc formed by platinum-decorated and CD-modified PDA NPs loaded with a ferrocene-appended ruthenium complex (RuFc) successfully delivered RuFc to the tumor sites, triggered by low pH, photothermal heating, and H_2_O_2_. The combined photodynamic and photothermal therapy overcame the hypoxic environment of tumors from platinum NP catalysis of O_2_·^−^ production from H_2_O_2_, vasodilation caused by photothermal heating sustaining the oxygen supplement, and photodynamic therapy exerted by RuFc occurring through the Fenton reaction (Figure 24) [192].

As CDs, pillar[6]arene can serve as a supramolecular host for Fc groups inside their hydrophobic cavity. A ternary pillar[6]arene-based supramolecular nanocatalyst (GO*x*@T-NPs) for CDT exhibited a high-efficiency catalytic ability to convert glucose, using GO, into H_2_O_2_ and gluconic acid, then to hydroxyl radicals via Fc-induced Fenton reaction, and to reduce the pH value inside cancer cells for significant enhancement of the CDT effect. It also showed sensitive glutathione-induced camptothecin (CPT) prodrug release capacity to further improve the efficiency of CDT. Hence, GO*x*@NPs possessed excellent ability to synergistically enhance the CDT [193].

### 8.4. MOFs

Metal–organic frameworks (MOFs) are promising nanomaterials with abundant micropores and large surface areas [194] that are useful for the delivery of therapeutics; they have been extensively developed in this direction [155] as well as in nanocatalysis [195] and gas storage and separation [196]. Ferrocene-containing MOFs are a recently well-developed area for various applications [197].

A cobalt-ferrocene nanoscale MOF (Co-Fc NMOF), synthesized using Co(OAc)_2_·4H_2_O and Fc(COOH)_2_ and isostructural to the reported Zn-Fc MOF, of ≈80 nm with the addition of PVP, presented high Fenton activity combined with glucose oxidase (GOx) as a cascade Fenton platform (Co-Fc@GOx) for enhanced tumor treatment, functioning as a biomimetic nanozyme [198]. In the tumor microenvironment, GOx delivered by Co-Fc NMOF was shown to catalyze endogenous glucose to H_2_O_2_ and gluconic acid, as indicated above with a pillar[6]arene nanomaterial reported in 2021 [193]. The Co-Fc NMOF, scrutinized using various cell lines by cell counting kit-8 (CCK-8) assay, showed the absence of clear in vitro toxicity to normal cells, including HUVEC, HL-7702, and HPNE cell lines, but displayed a dose-dependent cytotoxicity to 4T1 cancer cells due to the induction of intracellular hydroxyl radicals. Both in vitro and in vivo studies showed a remarkable ability of Co-Fc@GOx to inhibit cancer cells and tumor regression [198].

**Figure 25:**
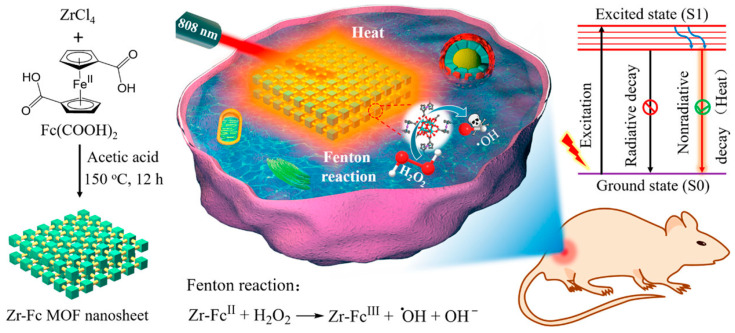
Synthesis of the Zr-Fc MOF nanosheet and illustration of synergetic PTT and Fenton reaction-based CDT. Reprinted with permission from Ref. [201]. Copyright 2020 American Chemical Society.

A ferrocene-containing MOF was prepared by reacting ZrCl_4_ with 1,1′-ferrocene di-carboxylic acid in acetic acid in precise amount (150 °C, 12 h). The resulting thin (16.4 nm thick) MOF (Zr-Fc MOF) nanosheet consisting of Zr_6_(μ_3_-O)_4_(μ_3_-OH)_4_ clusters as subunits [199] with Fc-di-carboxylate linkers [200,201] has been utilized for synergistic photothermal therapy (PTT) (photo conversion efficiency of 53% at 808 nm) and Fc-induced Fenton reaction-based hydroxyl radical generation as chemodynamic (CDT) therapy to cure cancer. The in situ generation of ROS is checked by ESR using 5,5-dimethyl-1-pyrroline N-oxide (DMPO) as the radical scavenger. UV–Vis absorbance spectroscopy using 5,5′-dithiobis(2-nitrobenzoic acid) (DNTB) as the chromogenic reagent showed that the GSH level decreased by ∼7% after laser irradiation because a hydroxyl radical generated under laser irradiation depleted GSH. In vitro, addition of 100 μg/mL Zr-Fc MOF nanosheet provoked almost 100% cell death. As in vitro, in vivo results with mice showed that PTT dominated the treatment while CDT was enhanced by PTT via the induction of localized hyperthermia. The resultant synergetic PTT and CDT treatment resulted in a very good anticancer therapeutic effect (Figure 25) [201].

Based on a similar 2D Zr-ferrocene (Zr-Fc)-MOF (Zr-Fc-MOF) nanosheet, Cu_2_O NPs have recently been grown by reducing Cu^2+^ by hydrazine hydrate in situ on the surface of Zr-Fc-MOF nanosheets to form the 2D Cu_2_O/Zr-Fc-MOF composite. The latter showed higher CD activity in the production of hydroxyl radicals via Fenton-like reaction compared with the pristine Zr-Fc-MOF nanosheets. The CD activity of the Cu_2_O/Zr-Fc-MOF composite was enhanced by photothermal effect using NIR laser (808 nm) irradiation toward efficient photothermally enhanced CD antibacterial therapy. In vitro, the repression of the Cu_2_O/Zr-Fc-MOF composite + H_2_O_2_ + NIR reached almost 100% on *Staphylococcus aureus* in pH 6.0 PBS buffer. After wound infection in mice with *Staphylococcus aureus,* the combination of PTT and CDT treatment accelerated wound healing that was almost complete on the seventh day without weight change in mice, indicating excellent biocompatibility and safety [13].

### 8.5. Dendrimers

**Figure 26:**
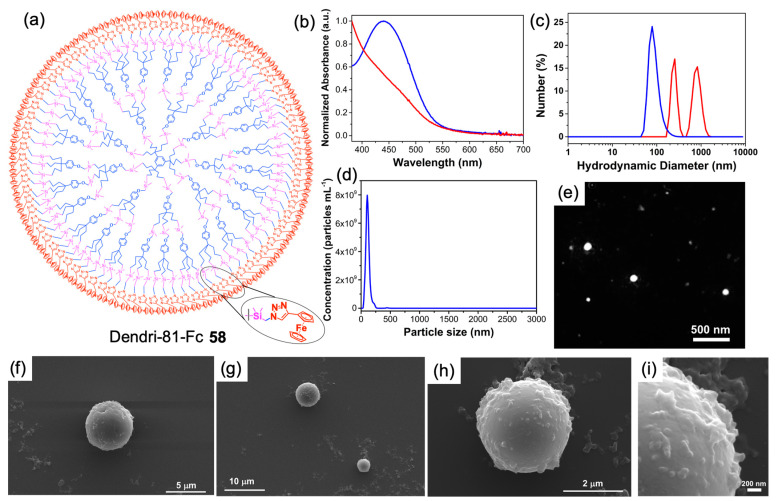
(**a**) Chemical structure of the 81-Fc dendrimer **58**; the inset shows the triazolylferrocenyl termini. (**b**) Absorption spectra of **58** normalized to the ferrocenyl band in CHCl_3_ (blue) and DMSO (red). (**c**) Hydrodynamic diameter of the self-assemblies formed by **58** in water obtained by DLS: nonfiltered solution (red) and filtered solution (blue). (**d**) Size distribution plot of the nanoassembly formed by **58** in aqueous medium (filtered solution) obtained by NTA. (**e**) Representative NTA video frame of the nanoparticles in solution. (**f**–**i**) SEM images of the self-assembled microparticles formed by **58**. Reproduced with permission from Ref. [221]. Copyright 2021 American Chemical Society.

Dendrimers are well-defined nanosized molecules with cauliflower- or tree-shape (dendrons) [149,202,203,204] that can encapsulate and deliver drugs [110,147,148,149,204,205,206,207], and a few recent publications have reported the production of hydroxyl radicals by the Fenton reaction inside dendrimers [208,209]. Ferrocene-terminated dendrimers that possess numerous ferrocenyl groups and fully reversible redox properties are candidates for the Fenton reaction and drug release [210,211,212,213]. Along these studies, it was noticed that large ferrocene dendrimers self-assembled into remarkably discrete regular-sized nanoparticles, just like atoms [213]. Aggregation-induced emission (AIE) from such assemblies was assigned in 2001 by Tang to restriction of intramolecular rotation inhibiting non-radiative decay [214], a concept extended inter alia to collective intra- and inter-chain n–*p* * interactions of heteroatoms [215,216]. It is understandable that large click dendrimers are subjected to such phenomena due to ferrocene termini inhibiting facile intra-branch movements. Indeed, Tomalia has recently associated dendrimer aggregation with a new photoluminescence phenomenon, referred to as non-traditional intrinsic luminescence (NTIL) [217,218]. Various dendritic nanomaterials are known to supramolecularly self-assemble into vesicles with luminescent properties relevant to NTIL [219,220]. The triazolylferrocenyl dendrimer **1** with 81 terminal triazolylferrocenyl groups was shown to self-assemble in aqueous medium into stable low-dispersity spherical-shaped vesosomes, emitting green NTI fluorescence (λ = 525 nm) upon excitation at 469 nm, including in biological medium, and presented cytotoxicity against cancer cells due to the numerous triazolylferrocenyl groups active in the Fenton reaction. Microscopy analysis revealed that formation of the vesosomes was not affected by the presence of enzymes, proteins, amino acids, antibiotics, lipids, hormones, vitamins, and salts present in the cell culture medium (Figure 26) [221].

The cytotoxicity of the vesosomes was evaluated against MCF-7 cells using the cell counting kit-8 (CCK-8) assay. MCF-7 cells were incubated for 24 and 48 h in the presence of the vesosomes. The 150.0 μM concentration tested resulted in 48% cell viability after 24 h (IC_50_: 168 μM of Fc groups or 2.1 μM of dendrimer), and incubation for 48 h resulted in 38% cell viability (IC_50_: 124 μM of Fc groups or 1.5 μM of dendrimer) [221].

The cytotoxicity of a new water-soluble dendrimer with 18 triazolylferrocenyl moieties and 18 free carboxylic acid groups was evaluated against MCF-7 (breast adenocarcinoma), HeLa (cervical cancer), PC3 (prostate adenocarcinoma), Caco-2 (colorectal cancer), and PNT2 (normal prostate cells) cell lines, and the assays showed that the dendrimer exhibited moderate anticancer activity and targeted cancer cells selectively over normal cells [222]. The variety of known and possible precise dendritic structures [157,158,159,203,204,217,223] is promising for the design of future nanodrugs.

A star-shaped and a dendritic tetranuclear ferrocenylamino complexes were evaluated against two colon cancer cell lines, the wild-type HT-29 and mutant DLD-1, after being treated with 20 μM of complexes and incubated for 24 h, followed by flow cytometry analysis. Moderate micromolar cytotoxic activity in both cell lines was reported, but the cellular uptake demonstrated a good correlation with early induction of apoptosis, and cellular treatment with these complexes increased the number of apoptotic cells [224].

## 9. Conclusions and Prospects

Due to its robustness in two oxidation states, a redox potential compatible with the Fenton reaction, its lipophilicity, barrel shape, and great possibilities of functionalization, ferrocene has been introduced into numerous drugs for various diseases. The pioneering discovery in 1996–1997 by Jaouen et al. of the anticancer drug ferroxifen adapted from the organic drug tamoxifen and the discovery by the Biot-Brossard group of the antimalarial drug ferroquin adapted from chloroquin resulted from full understanding and exploitation of these key properties. These two success stories in the biorganometallic/bioinorganic community have led to the development of a very large number of bioactive ferrocene-containing pharmacophores, including a number of heterobimetallic compounds, the second metal moiety being Ru, Pd, or Pt, in particular Pt moieties of the cisplatin group [10]. Several anticancer and antiparasitic compounds were often found to be also active against other diseases (antimicrobial and/or antifungal). Synthetic strategies of ferrocene introduction into pharmacophores involved either replacement of a phenyl (ferrocifens), alkyl (ferroquins), or heterocyclic ring of a known drug by a ferrocenyl group or attachment of the latter to a variety of bioactive compounds, natural products, peptides, steroids, betulins, etc. via a functional group. Electronic delocalization by ferrocenyl conjugation with styrenyl, triazole, piperazine, chalcone, etc. is favorable toward oxidation. For instance, ferrocenyl chalcones were also found to be DNA/BSA-interacting agents and inhibitors of DNA topoisomerase I and II activity [225].

The details of the Fenton reaction [226], which is responsible for cytotoxicity, indicate that the Fe^III/II^ redox potential plays a crucial role in the hydroxyl radical generation, and this flexibility does not seem to have been fully controlled and exploited. Other biological studies can include, as recently emphasized by Segur, the evaluation of effects of such ferrocene-containing pharmacophores on the apoptosis and penetration through the blood–brain barrier that is favored by the ferrocene lipophilicity (although nonspecific cellular impermeability should be avoided) [227].

Impaired brain iron homeostatic mechanisms are harmful to the brain due to iron-induced oxidative stress [228]. Along this line, ferrocenecarboxaldoxime was recently found to increase L-ferritin levels in the olfactory bulb, neocortex, pallidum, thalamus, corpus callosum, hippocampal CA3 regions, and substantia nigra [229].

Molecular docking studies have become useful in anticipating optimized structure for protein and DNA interaction with ferrocenyl conjugates, for instance, for anti-coronavirus activity, although no ferrocenyl drug has yet appeared in this case [230].

Another problem is that the endogenous H_2_O_2_ and the acidity in the tumor microenvironment (TME) are not quite sufficient to provide a very efficient Fenton reaction, which is responsible for slow cytotoxicity. The recent introduction of glucose oxidase into the magic bullets for drug delivery upon glucose ultimate oxidation to H_2_O_2_ and acid was reported to dramatically accelerate the Fenton reaction (that can even be stimulated by heat and/or light). Along this line, an alternative strategy involves host–guest supramolecular interaction with hydroxyl radical-consuming glutathione to provoke glutathione depletion and thereby elevate the available hydroxyl radical concentration [231].

For this purpose, nanomaterials, such as designed ferrocene-containing polymers, micelles, liposomes, dendritic vesosomes, cyclodextrin (or pillar-arenes), and metal–organic frameworks (MOFs), have been shown to be suitable vectors. This nanomaterial family could expand its utilization to anticancer, antiparasitic, antiviral, antibacterial and antifungal ferrocene conjugates. The use of such nanovectors involves the so-called chemodynamic therapy (CDT) [23]. With such a strategy, hydroxyl radicals are selectively delivered to the TME because, in normal cells, the pH is not low enough and the H_2_O_2_ concentration is not high enough for damaging hydroxyl-generating Fenton reaction. Nevertheless, such a selectivity will need to be carefully scrutinized in clinical studies for side effects.

The generation of hydroxyl radical leading to tumor destruction can also be generated by radiotherapy (X-ray) and other chemotherapeutic agents, such as doxorubicin, tirapazamine, bleomicin, and the antimalarial drug artimisinin, whose peroxide bridge is a source of hydroxyl radicals. Combined therapies, such as combined DCT and PTT or multiple drug treatment, significantly increase the chances of tumor death. In sum, ferrocene conjugates should hold a privileged place among metallodrugs in various areas of nanomedicine [232]. The diagnostic being essential to drug efficacy on time, a theranostic (i.e., therapy + diagnostic) [233] strategy should be adopted, for example, using gold NPs [163,164,165] or dendrimers [221]. Finally, given this increasing selectivity and efficacy against a variety of diseases of ferrocene conjugates, it is hoped that the pace of clinical trial will be accelerated toward a dramatic decrease of infected patients.
